# Polymorphism in a lincRNA Associates with a Doubled Risk of Pneumococcal Bacteremia in Kenyan Children

**DOI:** 10.1016/j.ajhg.2016.03.025

**Published:** 2016-05-26

**Authors:** The Kenyan Bacteraemia Study Group, Wellcome Trust Case Control Consortium 2 (WTCCC2), Anna Rautanen, Matti Pirinen, Tara C. Mills, Kirk A. Rockett, Amy Strange, Anne W. Ndungu, Vivek Naranbhai, James J. Gilchrist, Céline Bellenguez, Colin Freeman, Gavin Band, Suzannah J. Bumpstead, Sarah Edkins, Eleni Giannoulatou, Emma Gray, Serge Dronov, Sarah E. Hunt, Cordelia Langford, Richard D. Pearson, Zhan Su, Damjan Vukcevic, Alex W. Macharia, Sophie Uyoga, Carolyne Ndila, Neema Mturi, Patricia Njuguna, Shebe Mohammed, James A. Berkley, Isaiah Mwangi, Salim Mwarumba, Barnes S. Kitsao, Brett S. Lowe, Susan C. Morpeth, Iqbal Khandwalla, The Kilifi Bacteraemia Surveillance Group, Jenefer M. Blackwell, Elvira Bramon, Matthew A. Brown, Juan P. Casas, Aiden Corvin, Audrey Duncanson, Janusz Jankowski, Hugh S. Markus, Christopher G. Mathew, Colin N.A. Palmer, Robert Plomin, Stephen J. Sawcer, Richard C. Trembath, Ananth C. Viswanathan, Nicholas W. Wood, Panos Deloukas, Leena Peltonen, Thomas N. Williams, J. Anthony G. Scott, Stephen J. Chapman, Peter Donnelly, Adrian V.S. Hill, Chris C.A. Spencer

**Affiliations:** 1 Wellcome Trust Centre for Human Genetics, University of Oxford, Roosevelt Drive, Oxford OX3 7BN, UK; 2 Wellcome Trust Sanger Institute, Wellcome Trust Genome Campus, Hinxton, Cambridge CB10 1SA, UK; 3 Department of Paediatrics, University of Oxford, Oxford OX3 9DU, UK; 4 KEMRI-Wellcome Trust Research Programme, Kilifi 80108, Kenya; 5 Nuffield Department of Clinical Medicine, University of Oxford, Oxford OX3 9DU, UK; 6 Department of Infectious Disease Epidemiology, London School of Hygiene and Tropical Medicine, London WC1E 7HT, UK; 7 Cambridge Institute for Medical Research and Department of Pathology, University of Cambridge, Tennis Court Road, Cambridge CB2 1QP, UK; 8 Telethon Kids Institute, The University of Western Australia, Subiaco, WA 6008, Australia; 9 National Institute for Health Research (NIHR) Biomedical Research Centre for Mental Health at the South London and Maudsley National Health Service (NHS) Foundation Trust and Institute of Psychiatry, King’s College London, Denmark Hill, London SE5 8AF, UK; 10 Institute of Health and Biomedical Innovation, Queensland University of Technology, Translational Research Institute, Princess Alexandra Hospital, Brisbane, QLD 4102, Australia; 11 Farr Institute of Health Informatics, University College London, London NW1 2DA, UK; 12 Neuropsychiatric Genetics Research Group, Institute of Molecular Medicine, Trinity College Dublin, Dublin 2, Ireland; 13 Molecular and Physiological Sciences, The Wellcome Trust, London NW1 2BE, UK; 14 Associate Deans Office, John Bull Building, Peninsula School of Medicine and Dentistry, Plymouth PL6 8BU, UK; 15 Department of Neurology, University of Cambridge, Cambridge CB2 0QQ, UK; 16 Division of Genetics and Molecular Medicine, King’s College London, London SE1 9RT, UK; 17 Sydney Brenner Institute for Molecular Bioscience, University of Witwatersrand, Johannesburg 2193, South Africa; 18 Medical Research Institute, University of Dundee, Ninewells Hospital and Medical School, Dundee DD1 9SY, UK; 19 MRC Social, Genetic and Developmental Psychiatry Centre, Institute of Psychiatry, King’s College London, De Crespigny Park, London SE5 8AF, UK; 20 Department of Clinical Neurosciences, University of Cambridge, Addenbrooke’s Hospital, Hills Road, Cambridge CB2 0QQ, UK; 21 London School of Medicine and Dentistry, Queen Mary University of London, London E1 2AT, UK; 22 NIHR Biomedical Research Centre at Moorfields Eye Hospital NHSFT and UCL Institute of Ophthalmology, London EC1V 2PD, UK; 23 Department of Molecular Neuroscience, Institute of Neurology, Queen Square, London WC1N 3BG, UK; 24 Department of Medicine, Imperial College, London W21NY, UK; 25 INDEPTH Network, Accra, Ghana; 26 Oxford Centre for Respiratory Medicine, Churchill Hospital Site, Oxford University Hospitals, Oxford OX3 7LE, UK; 27 Department of Statistics, University of Oxford, Oxford OX1 3TG, UK; 28 The Jenner Institute, University of Oxford, Old Road Campus Research Building, Oxford OX3 7DQ, UK

## Abstract

Bacteremia (bacterial bloodstream infection) is a major cause of illness and death in sub-Saharan Africa but little is known about the role of human genetics in susceptibility. We conducted a genome-wide association study of bacteremia susceptibility in more than 5,000 Kenyan children as part of the Wellcome Trust Case Control Consortium 2 (WTCCC2). Both the blood-culture-proven bacteremia case subjects and healthy infants as controls were recruited from Kilifi, on the east coast of Kenya. *Streptococcus pneumoniae* is the most common cause of bacteremia in Kilifi and was thus the focus of this study. We identified an association between polymorphisms in a long intergenic non-coding RNA (lincRNA) gene (AC011288.2) and pneumococcal bacteremia and replicated the results in the same population (p combined = 1.69 × 10^−9^; OR = 2.47, 95% CI = 1.84–3.31). The susceptibility allele is African specific, derived rather than ancestral, and occurs at low frequency (2.7% in control subjects and 6.4% in case subjects). Our further studies showed AC011288.2 expression only in neutrophils, a cell type that is known to play a major role in pneumococcal clearance. Identification of this novel association will further focus research on the role of lincRNAs in human infectious disease.

## Introduction

Bacteremia is a common pathway in the progression to death from severe pneumonia, meningitis, and sepsis, which together account for an estimated 3 million deaths each year globally in children under the age of 5 years. Even in developed countries the mortality rate from bacteremia remains unacceptably high.^1^, ^2^, ^3^ The leading bacterial cause of death in young children worldwide is *Streptococcus pneumoniae* (pneumococcus), and 14.5 million episodes of serious pneumococcal disease occur in young children annually.^4^ A key question is why only a proportion of individuals develop invasive disease despite widespread exposure and asymptomatic carriage of bacteria. Host genetic factors play an important role in explaining inter-individual variation in susceptibility to different infectious diseases.^5^ However, the relevant genes for bacteremia susceptibility remain largely unknown.

To identify genetic correlates of bacteremia susceptibility, we conducted a genome-wide association study (GWAS) in Kenyan children, a population with a major disease burden,^6^ as a part of the Wellcome Trust Case Control Consortium 2 (WTCCC2). Bacteremia is a heterogeneous phenotype and immune responses and genetic variants affecting susceptibility are likely to be at least partially pathogen specific. We therefore focused on bacteremia caused by *S. pneumoniae*, the most common bacteria found in our study. In addition, all-cause bacteremia was analyzed in order to assess the possible role of genetic risk factors for bacteremia regardless of its etiology.

## Subjects and Methods

### Study Design

To identify host genetic determinants of susceptibility to invasive pneumococcal disease in African children, we performed a two-stage GWAS of pneumococcal bacteremia in 542 Kenyan children with culture-confirmed disease and 4,013 healthy control subjects. 429 case subjects and 2,677 control subjects were included in the discovery phase analysis, with 113 case subjects and 1,336 control subjects included in the replication analysis. To identify determinants of invasive bacterial disease irrespective of the pathogen, we further performed a GWAS of culture-confirmed all-cause bacteremia in the same population of Kenyan children (discovery phase, 1,536 case subjects; replication phase, 434 case subjects). Adopting a Bayesian framework, we considered evidence for shared effects at loci associated with pneumococcal disease and all-cause bacteremia, across pathogens commonly causing bacteremia in this population. Finally, we characterized disease-associated genetic variation identified in the study, analyzing tissue-specific expression of implicated transcripts in immune cell subsets. A detailed study workflow is described in Figure S1.

### Study Participants

All study participants were residents of Kilifi District on the coast of Kenya. Case subjects were recruited among children younger than 13 years of age who were admitted to Kilifi District Hospital (KDH) in Kenya between 1^st^ August 1998 and 30^th^ October 2010. Blood cultures were investigated from everyone admitted (unless they were admitted for elective procedures or because of minor accidents) using the BACTEC 9050 system. Children with bacteria present in their bloodstream were defined as case subjects (*Coryneforms* bacteria, *Bacillus* species, coagulase-negative *Staphylococcus*, *Staphylococcus saprophyticus*, and Viridans group *Streptococcus* were excluded as contaminants). The annual incidence of bacteremia in Kilifi between August 1998 and July 2002 was estimated to be 505 cases per 100,000 children who were less than 5 years of age,^6^ but the incidence has since decreased.^7^

Control subjects were selected from children born consecutively within the same Kilifi region between 1^st^ May 2006 and 30^th^ April 2008 and represent the case subjects closely in terms of sex, ethnic group, and geographic area of residence. Although the control individuals are part of a birth cohort study and thus aged less than 12 months at the time of recruitment to the study, we have been able to review their follow-up data in terms of development of bacteremia (n = 12), mortality (n = 49), etc. (See further demographic details of case and control subjects in Table S2.) Table S3 shows the distribution of the most common bacterial isolates identified from bacteremia case subjects in the discovery and replication sets. The final discovery set included 1,536 blood-culture-proven bacteremia case subjects (of whom 429 were pneumococcal) and 2,677 healthy infants as control subjects. Individuals in the replication set were enrolled during the end of the collection period and included 434 bacteremia case subjects (of whom 113 were pneumococcal) and 1,336 control subjects.

Ethical approval was granted by the Kenya Medical Research Institute (KEMRI) National Scientific Steering and Research Committees and the Oxford Tropical Research Ethics Committee (OXTREC). Informed consent was obtained from all subjects.

### DNA Sample Preparation

Genomic DNA was extracted at the Kenya Medical Research Institute (KEMRI)-Wellcome Trust Collaborative Programme in Kenya, using the QIAamp DNA blood mini kit (QIAGEN) and shipped to the Wellcome Trust Centre for Human Genetics, University of Oxford, for further processing. Genomic DNA was whole-genome amplified at the GeneService laboratory with GenomiPhi (GE HealthCare) scaled to amplify 40–50 μg of DNA. Quality of the whole-genome amplified DNA was assessed at the Wellcome Trust Sanger Institute as described elsewhere^8^ before genotyping.

### Genome-wide Genotyping and Quality Control

Whole-genome amplified samples from case and control subjects were genotyped on the genome-wide Affymetrix SNP 6.0 chip at the Affymetrix service laboratory. Genotypes were called with a modified version of the Chiamo software^9^ for all samples passing the Affymetrix laboratory quality control measures. Sample QC was performed as described elsewhere^8^ and details are provided in Table S1 and Figures S1–S3. Analysis of pairwise allele sharing identified 68 duplicate pairs and 6 triplicates (Figure S4). Phenotypic information suggests that the majority of these duplicate and triplicate individuals were unintentionally recruited to the study in Kenya more than once rather than being sample handling problems; therefore, one of each of the duplicate pairs or triplicates was included in the analysis (a case rather than a control subject was included in the analysis; otherwise the sample with a higher call rate was included). First-degree relatives (genome-wide IBD sharing probability > 0.4; 117 individuals) were removed from the main analysis. The following criteria were used to exclude 102,896 unreliable SNPs: minor allele frequency (MAF) < 1% (50,322 SNPs), info < 0.975 (53,419 SNPs), Hardy-Weinberg equilibrium p < 1 × 10^−20^ (18,288 SNPs), plate effect p < 1 × 10^−6^ (7,382 SNPs), and SNP missingness > 2% (34,430 SNPs). Genotyping cluster plots of each SNP with p < 1 × 10^−3^ were visually inspected using Evoker,^10^ and SNPs with poor cluster separation were removed. After sample and SNP QC, 1,536 case subjects and 2,677 control subjects were analyzed at 787,861 genotyped autosomal SNPs. Three main ethnicities—namely Chonyi, Giriama, and Kauma—were discernible with principal-components analysis (PCA) of the genome-wide data (Figure S5).

### Immunochip Genotyping

Approximately 2,000 SNPs out of the total 200,000 SNPs were selected to be included in the ImmunoChip array^11^ based on the initial association results of the bacteremia analyses. The replication set was genotyped with this array at the Wellcome Trust Sanger Institute. All the samples went through a similar QC process as described above for the discovery samples (Table S1) and 434 case subjects and 1,336 control subjects passed the QC. After excluding SNPs based on minor allele frequency < 1%, SNP call rate < 95% (<99% if MAF < 5%), and Hardy-Weinberg equilibrium p < 1 × 10^−10^, 143,100 SNPs remained for the further analyses. The same ethnicities were detectable by PCA in the replication sample set as in the discovery analysis (Figure S6). As the ImmunoChip genotyping was performed before the imputation, these genotypes were mainly utilized to account for population stratification and relatedness in the later replication analyses.

### Imputation and Association Analyses

We performed whole-genome imputation using the 1000 Genomes Phase I data as a reference panel. Genotypes were pre-phased using SHAPEIT^12^ before imputation with IMPUTE2.^13^ Only samples and SNPs passing the QC were included for pre-phasing and imputation. SNPs with potentially unreliable imputation were filtered out based on MAF (<2%), imputation info value (<0.8), and Hardy-Weinberg equilibrium (p < 1 × 10^−10^). 10,996,499 imputed autosomal SNPs that passed the QC were analyzed for additive and genotypic models using SNPTEST2,^14^ taking the imputed genotype uncertainty (frequentist score test) and the first two principal components (PCs) of genetic structure into account. The genomic control parameter λ for bacteremia overall and pneumococcal bacteremia after imputation and QC were 1.043 and 1.013, respectively (see the QQ plots in Figure S7). At associated SNPs, statistical tests were also performed using a linear mixed model that uses genome-wide data to model the pair-wise relatedness among the individuals.^15^

### Sequenom Replication and Confirmation of Imputation Accuracy

SNPs with p < 1 × 10^−5^ in the additive model or p < 5 × 10^−7^ in the genotypic model were directly genotyped in the discovery set to confirm imputation accuracy and in the replication sample set to confirm the associations using two Sequenom iPLEX assays. Five SNPs looked unreliable after inspection of the cluster plots, leaving 37 SNPs in the analysis (the cluster plot for the most significant SNP is shown in Figure S8). All of these SNPs had a call rate greater than 95% and the genotype distribution among controls obeyed Hardy-Weinberg equilibrium (p > 0.05). After removing the samples that were originally excluded from the discovery and Immunochip analyses, 102 and 80 samples were removed because of the low call rate (<80%) and 7 and 9 samples because of the mismatching gender from the first and second multiplexes, respectively. This left 1,514 case subjects (418 pneumococcal cases) and 2,642 control subjects in the discovery sample set and 407 case subjects (103 pneumococcal cases) and 1,333 control subjects in the replication analyses. Genotyping of these two iPlexes was performed at the Wellcome Trust Sanger Institute. The functional SNP rs334 in *HBB* failed the initial assay design, and was therefore genotyped separately using a Sequenom iPlex at the Wellcome Trust Centre for Human Genetics, University of Oxford. The QC measures described above were applied to these samples, leaving 1,360 case subjects and 2,644 control subjects in the discovery set and 389 case subjects and 1,312 control subjects in the replication set.

Only the samples that were included in either the discovery set or ImmunoChip replication set were included in the final analysis to allow inclusion of the first two PCs in logistic regression analysis using PLINK^16^ and to model the pair-wise relatedness in a linear mixed model. The combined statistics for the discovery and replication samples were obtained using fixed effects meta-analysis in GWAMA.^17^ The replication dataset had 80% power to detect an association (p < 0.05) with a common SNP (MAF 0.20) that has an effect size ≥ 1.3, but for more rare SNPs (MAF = 0.05), an effect size ≥ 1.54 was required (see Figure S9). Therefore, we did not have sufficient statistical power to reliably replicate associations with modest effect sizes.

### Approaches to Handle Relatedness

The SNPs chosen for replication were also analyzed via a linear mixed model^15^ that uses genome-wide data to model the pair-wise relatedness among the individuals, and which also included the first two PCs as covariates, to better account for relatedness and possible population structure within the sampled individuals. This was done by including all relatives and also by including only distantly related individuals (r < 0.2).

We further assessed whether the sample set with pneumococcal infection includes more pairs of close relatives than other bacteremia case subjects or than control subjects. This was assessed by comparing the observed number of relative pairs with estimated r > 0.025 among the pneumococcus case subjects to an equal-sized set of the rest of the case or control subjects that are matched with respect to manual clustering (Figure S11) by resampling 100,000 datasets.

### Bayesian Model Comparisons

To compare models of the similarity of effect across bacterial species at identified disease-associated loci, we took a Bayesian approach (for a similar approach, see Band et al.^18^ and Bellenguez et al.^19^). The likelihood function is based on multinomial regression with strata corresponding to the control subjects and each of the seven most common bacterial subgroups (Figure 2; Table S3). Case subjects infected with more than one of these seven different bacterial species (2.1% of case subjects) are included in the analysis for each group.

The parameters of interest are the genetic effect sizes (b_k_, k = 1,..,7) on a log-odds scale for each of the case cohorts. We first find maximum likelihood estimates (with the corresponding observed information matrix) by including two PCs as covariates in the model, and then compute approximate Bayes factors using a multivariate normal approximation to the likelihood and the prior. The models are defined by prior distributions on the parameters b_k_:

NULL: b_k_ = 0 for all k = 1,..,7, i.e., no effects, all case groups are like the control group.
SAME: b_k_ ∼N(0,1) and cor(b_i_,b_j_) = 1 for all pairs i ≠ j, i.e., each b_k_ is the same.
REL: b_k_ ∼N(0,1) and cor(b_i_,b_j_) = 0.96 for all pairs i ≠ j, i.e., b_i_ and b_j_ are correlated but not necessarily the same.

Additional models are defined after inspection of the observed association at each locus for each pathogen. Bacterial species hypothesized to be associated with a given locus are assumed to have the same non-zero effect with a prior of N(0,1), whereas for other pathogens the effect is 0.

### Quantification of lincRNA Expression in Primary Immune Cell Subsets

Previous reports suggest that AC011288.2 encodes a lincRNA and is expressed in white blood cells and placental tissue. To identify which leukocyte population this lincRNA is expressed in, we isolated monocytes, B cells, and natural killer (NK) cells from consenting healthy adult European-ancestry donors using magnetic activated cell sorting (MACS, Miltenyi), as previously described.^20^ In addition, we isolated granulocytes (predominantly neutrophils) using Polymorphoprep (Allere) according to the manufacturer’s instructions from eight individuals. The purity of cell subsets after cell separation was assessed by flow cytometry and was >90% in a representative sample. Viability after sorting was assessed by the Trypan Blue dye exclusion method and observed to be >95% in all cases. Total RNA was extracted with the RNeasy mini kit (QIAGEN) or TRIzol (Life Technologies) according to the manufacturer’s instruction (QIAGEN). Total RNA was quantified by Nanodrop and Bioanalyzer for a subset according to the manufacturers’ instruction (Bioanalyzer RNA 6000 Nano kit, Agilent).

To quantify levels of lincRNA expression, we performed quantitative real-time PCR (qPCR) using a relative quantification method. Beta-Actin (ACTB) was selected as a reference gene based on previous reports of its stable expression in neutrophils. Single-strand complementary DNA was synthesized by reverse transcription with the SuperScriptIII First-Strand Synthesis System (Invitrogen). Primers specific to each of the two reported transcripts for the lincRNA AC011288.2 gene were designed: AC011288.2-001 (for, 5′-GTCAGAAGCGGGGTTCAAAG-3′; rev, 5′-TTTAATTCTTGAGTTCTGCAGGC-3′) and AC011288.2-002 (for, 5′-GATGCTAAGCCTGGAAACCC-3′; rev, 5′-TCCAGCTTCTATTCCCAGAGG-3′). In addition we designed primers to AC006000.5 (for, 5′-ACTCCACGTCCCACAGATAC-3′; rev, 5′-TGACAGAGTGAGACCCTGTG-3′) but consistent with previous reports that observed no expression in leucocytes, we did not identify any individuals that expressed this transcript and do not describe it further. To avoid potential amplification of genomic DNA, primers were designed to span exons. qPCR was performed using SYBR Green Supermix (BioRad) on a CFX96 Real-Time PCR Detection System (Bio-Rad). Reactions were run in duplicate with 1 cycle at 95°C (10 min), followed by 42 cycles consisting of denaturation at 95°C (10 s), annealing at 58°C (20 s), and extension at 72°C (20 s). Detection of the fluorescent products was carried out at the end of the 72°C extension period. To confirm amplification specificity, the PCR products were subjected to a melting curve analysis and agarose gel electrophoresis. Detection of a fluorescent product after cycle 38 (Ct = 38) was considered evidence of expression beneath the confident detection limit based on careful inspection of the melting curves and agarose gel electrophoresis results. Therefore, if Ct values of greater than 38 were obtained, the Ct value was re-assigned to 38, so as to conservatively estimate the highest level of expression beneath the detectable level. On average, the Ct value for AC0011288.2-001 was 33 cycles in neutrophils. Relative gene transcript levels were determined by the [DELTA][C.sub.T] method expressed relative to ACTB. Comparisons of log10 transformed relative expression levels were made using a non-parametric Mann-Whitney test in GraphPad Prism.

## Results

### Genome-wide Association Results of Directly Genotyped Discovery and Immunochip Replication Sets

We identified several suggestive associations in the directly genotyped discovery data both in pneumococcal bacteremia and bacteremia overall analyses in Kenyan children (Table S4), but none of the SNPs reached established criteria for identifying novel associations (p < 5 × 10^−8^) in a combined analysis after replication.

### Genome-wide Association Results of Bacteremia Caused by *Streptococcus pneumoniae* after Imputation and Replication

After genome-wide imputation and quality control, nearly 10 million autosomal SNPs were included in the association analyses of pneumococcal bacteremia (Figure S10). In this analysis of 429 case and 2,677 control subjects, 17 SNPs in a single region on chromosome 7 were associated with disease at a level exceeding genome-wide significance (p < 5 × 10^−8^), with the peak of that association observed at rs140817150 (p imputed = 7.25 × 10^−9^; OR = 2.74) (Figures S10 and 1). This novel associated region includes two overlapping long intergenic non-coding RNA (lincRNA) genes: AC00600.5 and AC011288.2. The association at rs140817150 was confirmed by direct genotyping (p discovery = 3.58 × 10^−7^; OR = 2.39) and replication (p = 1.16 × 10^−3^; OR = 2.72), resulting in a combined OR estimate of 2.47 (95% CI 1.84–3.31) and p value of 1.69 × 10^−9^ (see Table S5 for a list of all suggestive associations in the analysis of pneumococcal bacteremia and Table S6 for the comprehensive list of associated SNPs in the chromosome 7 top associated region). Direct genotyping of the top imputed SNPs confirmed that imputation was generally accurate (average concordance between imputed and directly genotyped genotypes was 98.3%). In order to protect against spurious associations due to possible cryptic relatedness, the SNPs chosen for replication were also analyzed using the mixed model approach (rs140817150; p combined = 1.5 × 10^−10^, OR = 2.66; Table S7) and by stratifying individuals on the basis of genetic background across the main ethnic groups (rs140817150; p discovery = 1.5 × 10^−7^, OR = 2.54; Figure S11). By either approach, the evidence for association remained strong. The pneumococcal case subjects do not have more close relatives than the control subjects in any of the four ancestry groups (p ≥ 0.22). When compared to other case subjects, they show elevated levels of relatedness (p < 0.05) in group 1 only, which does not contribute strongly to the observed association signal (Figure S11). After conditioning on the top SNP, no associations were detected with p < 10^−4^ in the region.

### Genome-wide Association Results of Bacteremia Overall after Imputation and Replication

In addition to the analysis of a more homogeneous phenotype, pneumococcal bacteremia, all-cause bacteremia overall (Figure S12) was also analyzed. Table S8 summarizes loci with the strongest evidence of association in bacteremia overall after imputation, including the replication results. The only genome-wide significant (p < 5 × 10^−8^) association signal that replicated was under the genotypic model, which allows independent effects on risk for homozygotes and heterozygotes. It revealed a strong association at the previously identified *HBB* locus (rs113892119, p = 5.08 × 10^−13^). The region of association included the functional rs334 polymorphism (p = 1.33 × 10^−10^) that leads to the production of hemoglobin S (HbS)^21^ and is located 25.6 kb upstream from the most associating SNP rs113892119.

As previously described, rs334 was associated with susceptibility among homozygotes^22^ (HbSS versus HbAA; directly genotyped combined p = 2.66 × 10^−12^, OR = 4.9) and with protection from bacteremia among heterozygotes^7^ (HbAS versus HbAA; directly genotyped combined p = 4.67 × 10^−3^, OR = 0.77). These same effects are seen in the most common bacterial subgroups (Figure S13).

### Bayesian Model Comparison Results of rs140817150 and rs334 Associations with Common Causes of Bacteremia in Kenyan Children

Although the association with rs140817150 was discovered in the pneumococcal bacteremia analysis, we were able to utilize the all-cause bacteremia data at this locus to assess its effect on susceptibility to bacteremia caused by other species (Figure 2). To assess whether the data were consistent with the same effect among case subjects with different species of bacteremic pathogen, we compared models via a Bayesian approach (Figure 2). Assuming all models to be equally likely a priori, the most probable model is the one in which the susceptibility is confined to pneumococcus, *Acinetobacter* species, and *Haemophilus influenzae*. Removing the pneumococcal group, from which the association was ascertained, weakened the evidence for effect heterogeneity. The same effect in all subtypes was found to be the most probable model for rs334 association (heterozygote risk and homozygote protection) in *HBB* (Figure S13).

### lincRNA Expression in Primary Immune Cell Subsets

We assessed AC011288.2 RNA expression in the major leukocyte cell subsets and observed expression only in neutrophils. Expression levels were below the detection limit in monocytes, B cells, and natural killer (NK) cells (Figure 3). To verify that expression of this transcript is constitutive in neutrophils, we measured expression in an additional 75 donors, recruited in a separate study,^23^ and observed detectable expression in all 75 donors. We did not observe AC00600.5 expression in any leukocyte subsets.

## Discussion

We report here a GWAS of bacteremia susceptibility, which is one of the few large-scale GWASs conducted in an African population to date. We identified an association between polymorphisms in two overlapping long intergenic non-coding RNA (lincRNA) genes (AC00600.5 and AC011288.2) and pneumococcal bacteremia, the most common cause of bacteremia in our study set. Although immune responses and genetic variants affecting bacteremia susceptibility are likely to be at least partially pathogen specific, we also analyzed the bacteremia overall dataset to identify more universal risk factors. The only genome-wide significant hit for bacteremia overall was in a previously reported gene, *HBB*, including the well-known rs334 polymorphism associated with the production of sickle hemoglobin.^21^

**Figure 1:**
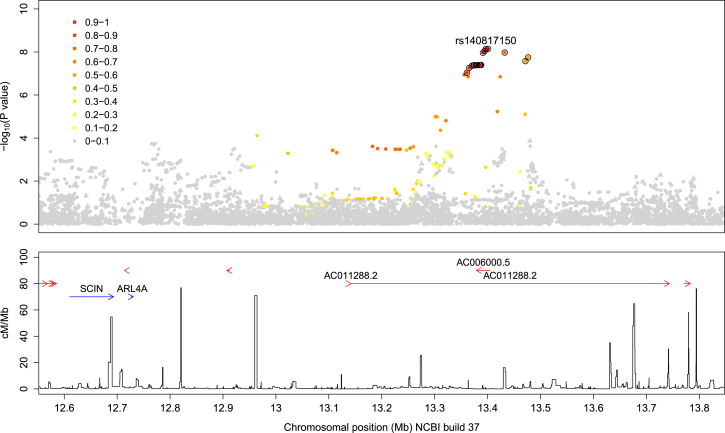
Signal of Association around rs140817150 in the Discovery Analysis of Pneumococcal Bacteremia Imputed SNPs are shown as circles and directly genotyped SNPs as triangles with colors indicating the correlation (r^2^ in 1000 Genomes data) with rs140817150. A set of SNPs that contains the causal SNP with greater than 95% probability is ringed with circles. Annotated genes (blue) and lincRNAs (red) are shown in the bottom panel along with the fine-scale recombination rate.

**Figure 2:**
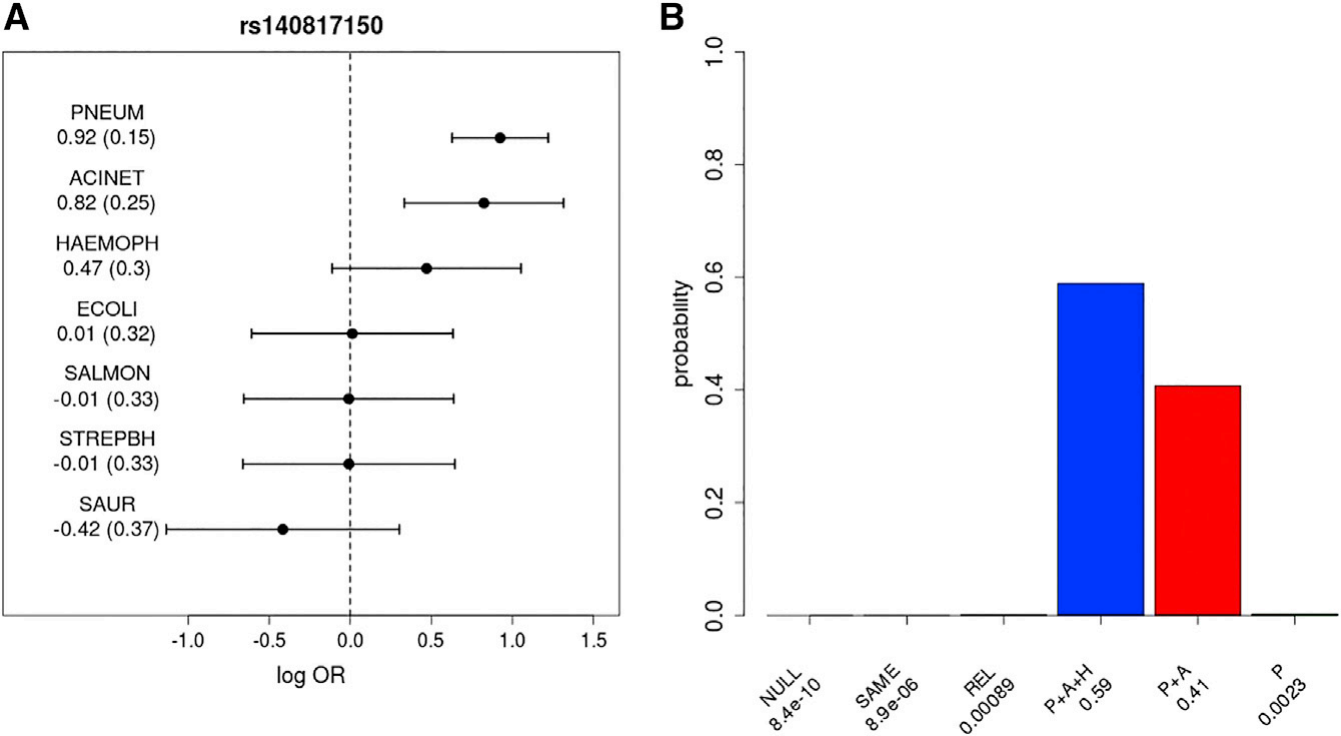
rs140817150 lincRNA Association with the Main Bacterial Infections (A) Log transformed combined odds ratios and 95% confidence intervals of directly genotyped discovery and replication samples. The dotted line represents the log OR of 0 (OR of 1; no difference between case and control subjects). The values of point estimates and standard errors (in parentheses) are also given. Bacterial infection abbreviations are as follows: PNEUM, *Streptococcus pneumoniae* (pneumococcus); ACINET, *Acinetobacter* species; HAEMOPH, *Haemophilus influenzae*; ECOLI, *Escherichia coli*; SALMON, *Salmonella* (non-typhoidal); STREPBH, *Streptococcus beta hemolytic*; SAUR, *Staphylococcus aureus*. (B) The posterior probabilities on the models of association: no effect in any subtype (NULL), same effect in all subtypes (SAME), related effects across subtypes (REL), or the same non-zero effect only in PNEUM, ACINET, and HAEMOPH (P+A+H), in PNEUM and ACINET (P+A), or in PNEUM (P). (See Subjects and Methods.) Models are a priori assumed to be equally likely. Bayes factors, which compare the evidence (marginal likelihood) between any pair of models, can be calculated as the ratio of the posterior probability assigned to each model as reported under each bar of the plot.

**Figure 3:**
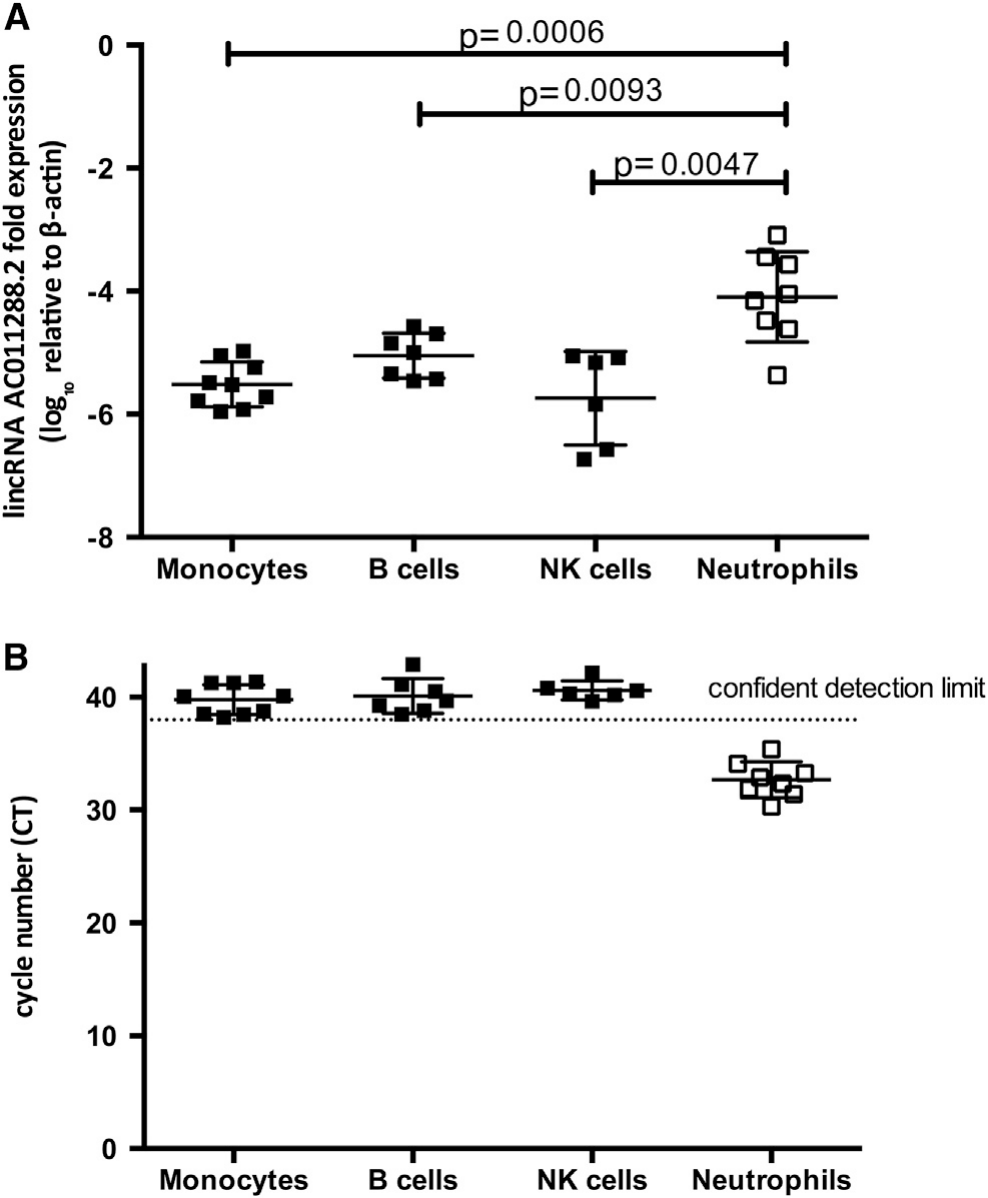
lincRNA AC011288.2 Expression Measured in Neutrophils, Monocytes, B Cells, and NK Cells (A) Quantitative PCR of AC011288.2-002 in primary leucocyte subsets. To conservatively estimate the highest level of expression beneath the detectable level, CT values greater than 38 were re-assigned to 38, and normalized to β-actin expression. p values denote the significance of the relative expression levels of AC011288.2-001 in neutrophils compared to other cell types (Mann-Whitney test). (B) Unadjusted cycle number of amplification is shown. Filled squares denote detection of a fluorescent product after cycle 38 (Ct = 38), the limit of confident detection being based on careful inspection of the melting curves. Similar results for AC011288.2-001 were obtained (data not shown).

The lincRNA risk allele at rs140817150 is derived (as reported on dbSNP) rather than ancestral, its frequency is low (2.7% in control subjects, 6.4% in pneumococcal bacteremia case subjects), and according to the 1000 Genomes project data (phase 3), it is polymorphic only in African populations. Consistent with the local recombination landscape and with the expectation that low-frequency derived alleles are relatively young, SNPs in linkage disequilibrium with rs140817150 extend over 500 kb (Figure 1). However, Bayesian analysis of the region of association^24^ in the imputed data suggests there is greater than 95% probability that one of the most associated SNPs (circled in Figure 1) is the causal variant, assuming there is a single causal variant, and it is imputed accurately in our dataset.

The association peak is located in the introns of two separate long intergenic non-coding RNAs (lincRNAs), annotated as AC011288.2 and AC006000.5. The importance of lincRNAs as key regulators of gene expression has only recently been recognized.^25^, ^26^, ^27^ It has been estimated that the human genome includes at least 10,000 lincRNAs but only a fraction of these has a known function.^25^, ^26^, ^27^ A recent study aiming to catalog the function of more than 8,000 human lincRNAs reported that lincRNA expression is significantly more tissue specific than expression of protein-coding genes.^28^ AC006000.5 is listed in the catalog but it is not expressed in any of the studied tissues, whereas AC011288.2 is reported to be expressed only in placenta and white blood cells out of 24 different tissues and cell lines studied. We assessed AC011288.2 RNA expression in leukocyte cell subsets and observed expression only in neutrophils, a cell type that is known to play a major role in pneumococcal clearance.^29^, ^30^ These results provide an important direction for future functional investigations. Neutrophils express many antimicrobial peptides and proteins that confer both universal and pathogen-specific host response,^31^, ^32^ and it has been shown that absolute neutrophil count is an independent predictor of pneumococcal bacteremia in febrile children.^33^

The closest protein-coding genes surrounding the association signal are *ARL4A* and *ETV1* but there is no evidence that the associating lincRNAs regulate these two genes. However, data from a previous expression quantitative trait locus (eQTL) study^20^ suggest that there are some SNPs in the associating region that function as eQTLs in monocytes (rs1432496) and B cells (rs2568633) for *PHF14* (PHD finger protein 14), a transcription factor that downregulates *PDGFRα* expression.^34^ However, neither is correlated with our most-associated SNP (r^2^ < 0.01 in 1000 Genomes data). Although the role of lincRNAs in human infections is unknown, recent mouse studies have indicated that some lincRNAs can act in immune cells to regulate host susceptibility to bacterial and viral infections.^35^, ^36^

Using the GWAS approach, we have identified an association between a genetic variation in a lincRNA gene and pneumococcal bacteremia. Furthermore, we have confirmed a previously reported association between *HBB* and bacteremia overall,^22^ with homozygotes associated with strong susceptibility but heterozygotes associated with protection. At both associated loci, the disease-associated alleles are rare in individuals without African ancestry (monomorphic in 1000 Genomes Project data in other than African populations) and exert a large effect on the likelihood of developing bacteremia. These associations have not been reported by earlier GWASs of related phenotypes, which is unsurprising because the populations under study have been of European decent and differences in phenotypes are still substantial.^37^, ^38^ The reported SNPs in *FER* (MIM: 176942) that was recently associated with outcome from sepsis due to pneumonia^37^ or in *CFH* (MIM: 134370) and *CFHR3* (MIM: 605336) that have been associated with meningococcal disease^38^ did not show any evidence of association in the current study (p > 0.05). Given the likely importance of host-pathogen molecular interactions in bacteremia susceptibility, it is plausible that the effect of a risk allele will be dependent on bacterial species. Our data on the lincRNA locus provide initial evidence for this at the bacterial species level, and motivate approaches that stratify host genetic associations by pathogen species, serotype, or genotype. Understanding the molecular mechanisms leading to the doubled risk of pneumococcal bacteremia associated with this allele could provide new clues in the pressing search for new therapeutic targets.

## Consortia

The Kenyan Bacteraemia study group consists of the following individuals. Principal investigators are Adrian V.S. Hill (Chair), Thomas N. Williams, J. Anthony G. Scott, and Stephen J. Chapman. Key personnel are Anna Rautanen, Tara C. Mills, Kirk A. Rockett, Anne W. Ndungu, Vivek Naranbhai, Alex W. Macharia, Sophie Uyoga, Carolyne Ndila, Neema Mturi, Patricia Njuguna, Shebe Mohammed, James A. Berkley, Isaiah Mwangi, Salim Mwarumba, Barnes S. Kitsao, Brett S. Lowe, Susan C. Morpeth, and Iqbal Khandwalla. The Kilifi DNA Extraction Group members are Alex W. Macharia, Sophie Uyoga, Herbert Opi, Carolyne Ndila, Emily Nyatichi, Prophet Ingosi, Barnes Kitsao, Clement Lewa, Johnstone Makale, Adan Mohamed, Kenneth Magua, Mary Njoroge, Gideon Nyutu, Ruth Mwarabu, Metrine Tendwa, and Thomas N. Williams. The Kilifi Bacteraemia Surveillance Group consists of the following individuals: Ismail Ahmed, Samuel Akech, Alexander Balo Makazi, Mohammed Bakari Hajj, Andrew Brent, Charles Chesaro, Hiza Dayo, Richard Idro, Patrick Kosgei, Kathryn Maitland, Kevin Marsh, Laura Mwalekwa, Shalton Mwaringa, Charles Newton, Mwanajuma Ngama, Allan Pamba, Norbert Peshu, Anna Seale, Alison Talbert, and Thomas N. Williams.

Wellcome Trust Case Control Consortium 2 consists of the following individuals. Management committee members are Peter Donnelly (Chair), Ines Barroso (Deputy Chair), Jenefer M. Blackwell, Elvira Bramon, Matthew A. Brown, Juan P. Casas, Aiden Corvin, Panos Deloukas, Audrey Duncanson, Janusz Jankowski, Hugh S. Markus, Christopher G. Mathew, Colin N.A. Palmer, Robert Plomin, Anna Rautanen, Stephen J. Sawcer, Richard C. Trembath, Ananth C. Viswanathan, and Nicholas W. Wood. Data and Analysis Group members are Chris C.A. Spencer, Gavin Band, Céline Bellenguez, Colin Freeman, Garrett Hellenthal, Eleni Giannoulatou, Matti Pirinen, Richard D. Pearson, Amy Strange, Zhan Su, Damjan Vukcevic, and Peter Donnelly. DNA, Genotyping, Data QC, and Informatics Group members are Cordelia Langford, Sarah E. Hunt, Sarah Edkins, Rhian Gwilliam, Hannah Blackburn, Suzannah J. Bumpstead, Serge Dronov, Matthew Gillman, Emma Gray, Naomi Hammond, Alagurevathi Jayakumar, Owen T. McCann, Jennifer Liddle, Simon C. Potter, Radhi Ravindrarajah, Michelle Ricketts, Matthew Waller, Paul Weston, Sara Widaa, Pamela Whittaker, Ines Barroso, and Panos Deloukas. Publications Committee members are Christopher G. Mathew (Chair), Jenefer M. Blackwell, Matthew A. Brown, Aiden Corvin, and Chris C.A. Spencer.

## Accession Numbers

Genotype data will be made available by the WTCCC access process, via the European Genotype Archive under the accession number EGAS00001001756.

## Web Resources

1000 Genomes, http://www.1000genomes.org

European Genome-phenome Archive (EGA), https://www.ebi.ac.uk/ega

OMIM, http://www.omim.org/

## Supplemental Data

Document S1. Figures S1–S13 and Tables S1–S8

Document S2. Article plus Supplemental Data

## References

[1] Lillie P.J., Allen J., Hall C., Walsh C., Adams K., Thaker H., Moss P., Barlow G.D. (2013). Long-term mortality following bloodstream infection. Clin. Microbiol. Infect..

[2] Wyllie D.H., Crook D.W., Peto T.E. (2006). Mortality after *Staphylococcus aureus* bacteraemia in two hospitals in Oxfordshire, 1997-2003: cohort study. BMJ.

[3] Laupland K.B., Svenson L.W., Gregson D.B., Church D.L. (2011). Long-term mortality associated with community-onset bloodstream infection. Infection.

[4] O’Brien K.L., Wolfson L.J., Watt J.P., Henkle E., Deloria-Knoll M., McCall N., Lee E., Mulholland K., Levine O.S., Cherian T., Hib and Pneumococcal Global Burden of Disease Study Team (2009). Burden of disease caused by *Streptococcus pneumoniae* in children younger than 5 years: global estimates. Lancet.

[5] Sørensen T.I., Nielsen G.G., Andersen P.K., Teasdale T.W. (1988). Genetic and environmental influences on premature death in adult adoptees. N. Engl. J. Med..

[6] Berkley J.A., Lowe B.S., Mwangi I., Williams T., Bauni E., Mwarumba S., Ngetsa C., Slack M.P., Njenga S., Hart C.A. (2005). Bacteremia among children admitted to a rural hospital in Kenya. N. Engl. J. Med..

[7] Scott J.A., Berkley J.A., Mwangi I., Ochola L., Uyoga S., Macharia A., Ndila C., Lowe B.S., Mwarumba S., Bauni E. (2011). Relation between falciparum malaria and bacteraemia in Kenyan children: a population-based, case-control study and a longitudinal study. Lancet.

[8] Barrett J.C., Lee J.C., Lees C.W., Prescott N.J., Anderson C.A., Phillips A., Wesley E., Parnell K., Zhang H., Drummond H., UK IBD Genetics Consortium, Wellcome Trust Case Control Consortium 2 (2009). Genome-wide association study of ulcerative colitis identifies three new susceptibility loci, including the HNF4A region. Nat. Genet..

[9] Consortium W.T.C.C., Wellcome Trust Case Control Consortium (2007). Genome-wide association study of 14,000 cases of seven common diseases and 3,000 shared controls. Nature.

[10] Morris J.A., Randall J.C., Maller J.B., Barrett J.C. (2010). Evoker: a visualization tool for genotype intensity data. Bioinformatics.

[11] Cortes A., Brown M.A. (2011). Promise and pitfalls of the Immunochip. Arthritis Res. Ther..

[12] Delaneau O., Marchini J., Zagury J.F. (2012). A linear complexity phasing method for thousands of genomes. Nat. Methods.

[13] Howie B.N., Donnelly P., Marchini J. (2009). A flexible and accurate genotype imputation method for the next generation of genome-wide association studies. PLoS Genet..

[14] Marchini J., Howie B. (2010). Genotype imputation for genome-wide association studies. Nat. Rev. Genet..

[15] Pirinen M., Donnelly P., Spencer C.C. (2013). Efficient computation with a linear mixed model on large-scale data sets with applications to genetic studies. Ann. Appl. Stat..

[16] Purcell S., Neale B., Todd-Brown K., Thomas L., Ferreira M.A., Bender D., Maller J., Sklar P., de Bakker P.I., Daly M.J., Sham P.C. (2007). PLINK: a tool set for whole-genome association and population-based linkage analyses. Am. J. Hum. Genet..

[17] Mägi R., Morris A.P. (2010). GWAMA: software for genome-wide association meta-analysis. BMC Bioinformatics.

[18] Band G., Le Q.S., Jostins L., Pirinen M., Kivinen K., Jallow M., Sisay-Joof F., Bojang K., Pinder M., Sirugo G., Malaria Genomic Epidemiology Network (2013). Imputation-based meta-analysis of severe malaria in three African populations. PLoS Genet..

[19] Bellenguez C., Bevan S., Gschwendtner A., Spencer C.C., Burgess A.I., Pirinen M., Jackson C.A., Traylor M., Strange A., Su Z., International Stroke Genetics Consortium (ISGC), Wellcome Trust Case Control Consortium 2 (WTCCC2) (2012). Genome-wide association study identifies a variant in HDAC9 associated with large vessel ischemic stroke. Nat. Genet..

[20] Fairfax B.P., Makino S., Radhakrishnan J., Plant K., Leslie S., Dilthey A., Ellis P., Langford C., Vannberg F.O., Knight J.C. (2012). Genetics of gene expression in primary immune cells identifies cell type-specific master regulators and roles of HLA alleles. Nat. Genet..

[21] Ingram V.M. (1957). Gene mutations in human haemoglobin: the chemical difference between normal and sickle cell haemoglobin. Nature.

[22] Williams T.N., Uyoga S., Macharia A., Ndila C., McAuley C.F., Opi D.H., Mwarumba S., Makani J., Komba A., Ndiritu M.N. (2009). Bacteraemia in Kenyan children with sickle-cell anaemia: a retrospective cohort and case-control study. Lancet.

[23] Naranbhai V., Fairfax B.P., Makino S., Humburg P., Wong D., Ng E., Hill A.V., Knight J.C. (2015). Genomic modulators of gene expression in human neutrophils. Nat. Commun..

[24] Maller J.B., McVean G., Byrnes J., Vukcevic D., Palin K., Su Z., Howson J.M., Auton A., Myers S., Morris A., Wellcome Trust Case Control Consortium (2012). Bayesian refinement of association signals for 14 loci in 3 common diseases. Nat. Genet..

[25] Derrien T., Johnson R., Bussotti G., Tanzer A., Djebali S., Tilgner H., Guernec G., Martin D., Merkel A., Knowles D.G. (2012). The GENCODE v7 catalog of human long noncoding RNAs: analysis of their gene structure, evolution, and expression. Genome Res..

[26] Djebali S., Davis C.A., Merkel A., Dobin A., Lassmann T., Mortazavi A., Tanzer A., Lagarde J., Lin W., Schlesinger F. (2012). Landscape of transcription in human cells. Nature.

[27] Lee J.T. (2012). Epigenetic regulation by long noncoding RNAs. Science.

[28] Cabili M.N., Trapnell C., Goff L., Koziol M., Tazon-Vega B., Regev A., Rinn J.L. (2011). Integrative annotation of human large intergenic noncoding RNAs reveals global properties and specific subclasses. Genes Dev..

[29] Gingles N.A., Alexander J.E., Kadioglu A., Andrew P.W., Kerr A., Mitchell T.J., Hopes E., Denny P., Brown S., Jones H.B. (2001). Role of genetic resistance in invasive pneumococcal infection: identification and study of susceptibility and resistance in inbred mouse strains. Infect. Immun..

[30] Brinkmann V., Reichard U., Goosmann C., Fauler B., Uhlemann Y., Weiss D.S., Weinrauch Y., Zychlinsky A. (2004). Neutrophil extracellular traps kill bacteria. Science.

[31] Cederlund A., Agerberth B., Bergman P. (2010). Specificity in killing pathogens is mediated by distinct repertoires of human neutrophil peptides. J. Innate Immun..

[32] Nguyen Q.T., Nguyen T.H., Ju S.A., Lee Y.S., Han S.H., Lee S.C., Kwon B.S., Yu R., Kim G.Y., Lee B.J., Kim B.S. (2013). CD137 expressed on neutrophils plays dual roles in antibacterial responses against Gram-positive and Gram-negative bacterial infections. Infect. Immun..

[33] Kuppermann N., Fleisher G.R., Jaffe D.M. (1998). Predictors of occult pneumococcal bacteremia in young febrile children. Ann. Emerg. Med..

[34] Kitagawa M., Takebe A., Ono Y., Imai T., Nakao K., Nishikawa S., Era T. (2012). Phf14, a novel regulator of mesenchyme growth via platelet-derived growth factor (PDGF) receptor-α. J. Biol. Chem..

[35] Gomez J.A., Wapinski O.L., Yang Y.W., Bureau J.F., Gopinath S., Monack D.M., Chang H.Y., Brahic M., Kirkegaard K. (2013). The NeST long ncRNA controls microbial susceptibility and epigenetic activation of the interferon-γ locus. Cell.

[36] Carpenter S., Aiello D., Atianand M.K., Ricci E.P., Gandhi P., Hall L.L., Byron M., Monks B., Henry-Bezy M., Lawrence J.B. (2013). A long noncoding RNA mediates both activation and repression of immune response genes. Science.

[37] Rautanen A., Mills T.C., Gordon A.C., Hutton P., Steffens M., Nuamah R., Chiche J.D., Parks T., Chapman S.J., Davenport E.E., ESICM/ECCRN GenOSept Investigators (2015). Genome-wide association study of survival from sepsis due to pneumonia: an observational cohort study. Lancet Respir. Med..

[38] Davila S., Wright V.J., Khor C.C., Sim K.S., Binder A., Breunis W.B., Inwald D., Nadel S., Betts H., Carrol E.D., International Meningococcal Genetics Consortium (2010). Genome-wide association study identifies variants in the CFH region associated with host susceptibility to meningococcal disease. Nat. Genet..

