# Supplementary material for: Polymorphism in a lincRNA Associates with a Doubled Risk of Pneumococcal Bacteremia in Kenyan Children
Source: Am J Hum Genet. 2016 May 26;98(6):1092–100. doi: 10.1016/j.ajhg.2016.03.025 (PMC4908194; doi:10.1016/j.ajhg.2016.03.025)
Supplement: Document S1. Figures S1–S13 and Tables S1–S8 [file mmc1.pdf]

The American Journal of Human Genetics, Volume *98*

Supplemental Data

Polymorphism in a lincRNA Associates with a Doubled Risk of Pneumococcal Bacteremia in Kenyan Children

The Kenyan Bacteraemia Study Group, Wellcome Trust Case Control Consortium 2 (WTCCC2), Anna Rautanen, Matti Pirinen, Tara C. Mills, Kirk A. Rockett, Amy Strange, Anne W. Ndungu, Vivek Naranbhai, James J. Gilchrist, Céline Bellenguez, Colin Freeman, Gavin Band, Suzannah J. Bumpstead, Sarah Edkins, Eleni Giannoulatou, Emma Gray, Serge Dronov, Sarah E. Hunt, Cordelia Langford, Richard D. Pearson, Zhan Su, Damjan Vukcevic, Alex W. Macharia, Sophie Uyoga, Carolyne Ndila, Neema Mturi, Patricia Njuguna, Shebe Mohammed, James A. Berkley, Isaiah Mwangi, Salim Mwarumba, Barnes S. Kitsao, Brett S. Lowe, Susan C. Morpeth, Iqbal Khandwalla, The Kilifi Bacteraemia Surveillance Group, Jenefer M. Blackwell, Elvira Bramon, Matthew A. Brown, Juan P. Casas, Aiden Corvin, Audrey Duncanson, Janusz Jankowski, Hugh S. Markus, Christopher G. Mathew, Colin N.A. Palmer, Robert Plomin, Stephen J. Sawcer, Richard C. Trembath, Ananth C. Viswanathan, Nicholas W. Wood, Panos Deloukas, Leena Peltonen, Thomas N. Williams, J. Anthony G. Scott, Stephen J. Chapman, Peter Donnelly, Adrian V.S. Hill, and Chris C.A. Spencer

Supplemental Figures

Figure S1: Bacteremia GWAS workflow

Genomic DNA whole-genome amplified with GenomiPhi kit

Discovery GWAS: ~2000 cases and ~3000 controls submitted to Affymetrix for genotyping after passing the sample QC performed at Sanger

- Number of excluded samples based on QC: 581
  - Failed at Affymetrix: 92
  - Call rate/heterozygosity: 182 (Figure S2)
  - Discrepancy between Sequenom and Affymetrix genotyping: 17 (5 that otherwise passed the QC)
  - Channel: 14
  - Gender discrepancy or unknown reported gender: 110 (32 that otherwise passed the QC)
  - Population outlier (PCA): 186 (Figure S3)
  - Duplicate individuals: 103 (of which 16 are intentional for QC purposes)
  - Relatedness (Siblings removed but more distant relatives kept in the analysis, pi_hat>0.4): 117
- Exclusion based on phenotype: 130 (See Table S1 for further details)
- Analysis of 1536 cases and 2677 controls using the first two principal components as covariates (PLINK)
- 2000 SNPs selected for Immunochip replication:
  - 1000: SNPs chosen based on bacteremia overall analysis
  - 1000: SNPs chosen based on different species of bacteremic pathogen

Immunochip replication: ~500 cases and ~1500 controls

- Number of excluded samples based on QC:212
  - Call rate/heterozygosity: 84
  - Discrepancy between Sequenom and ImmunoChip genotyping: 8
  - Channel: 4
  - Gender discrepancy: 5 (0 that would have otherwise passed the QC)
  - Population outlier (PCA): 51
  - Duplicate individual: 6
  - Duplicate individual between GWAS and Immunochip: 29
  - Relatedness (Siblings removed but more distant relatives kept in the analysis, pi_hat>0.4): 29
- Exclusion based on phenotype: 37 (See Table S1 for further details)
- Analysis of 434 cases and 1336 controls using the first two principal components as covariates (PLINK)
- No Genome-wide significant hits with bacteremia overall or in bacterial subgroups in the combined analysis

Imputation of the discovery set using individuals in the 1000 Genomes Project as a reference panel:

- Phasing with SHAPEIT (QCd set of samples and SNPs)
- Imputation with IMPUTE2
- Association analysis with SNPTEST2 using the score method and additive and genotypic models of association
  - SNPs with Info<0.8 and MAF<0.01 excluded
- The most promising associations selected for direct genotyping with Sequenom

Direct Sequenom genotyping of the discovery and replication samples: 37 SNPs in 3 iPLEX assays

- Samples excluded based on a call rate of <80% and mismatching gender
- Discovery and replication samples analyzed separately
    - Discovery: 1514 cases and 2642 controls that passed the GWAS QC earlier
    - Discovery of rs334 (*HBB*): 1360 cases and 2644 controls
    - Replication: 407 cases and 1333 controls that earlier passed the ImmunoChip QC
    - Replication of rs334 (*HBB*): 389 cases and 1312 controls that passed the ImmunoChip QC
    - Two loci reached genome-wide significance: *HBB* in bacteremia overall (genotypic model) and a lincRNA in the pneumococcal subgroup (allelic model)

- Mixed Model analysis to better account for relatedness and underlying population structure
    - Siblings and more distantly related individuals included in the analysis:
        - Discovery: 1519 cases and 2688 controls
        - Replication: 408 cases and 1360 controls
    - All related individuals (r>0.2) excluded from the analysis:
        - Discovery: 1476 cases and 2543 controls
        - Replication: 404 cases and 1311 controls

Figure S2. Sample exclusions based on call rate (x-axis) and heterozygosity (y-axis). Excluded samples are shown in red.

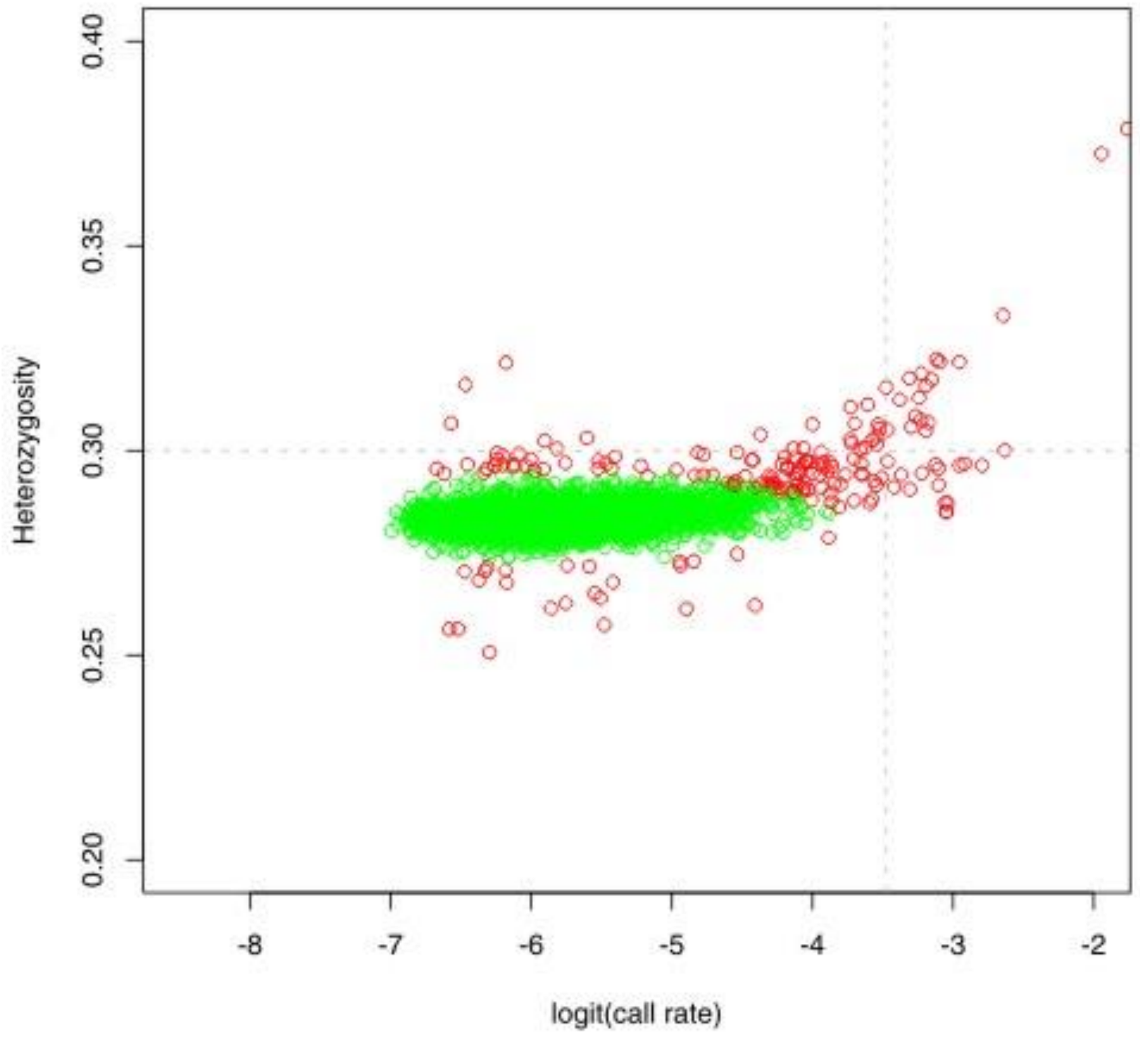

Figure S3. Principal components analysis for bacteremia cases and controls (A) analyzed together with HapMap individuals and (B) zoomed into the bacteremia cluster to show the population outlier exclusions (red). BS = bacteremia susceptibility; CEU = Utah residents with Northern and Western European ancestry from the CEPH collection; JPT = Japanese in Tokyo, Japan; CHB = Han Chinese in Beijing, China; YRI = Yoruba in Ibadan, Nigeria.

A)

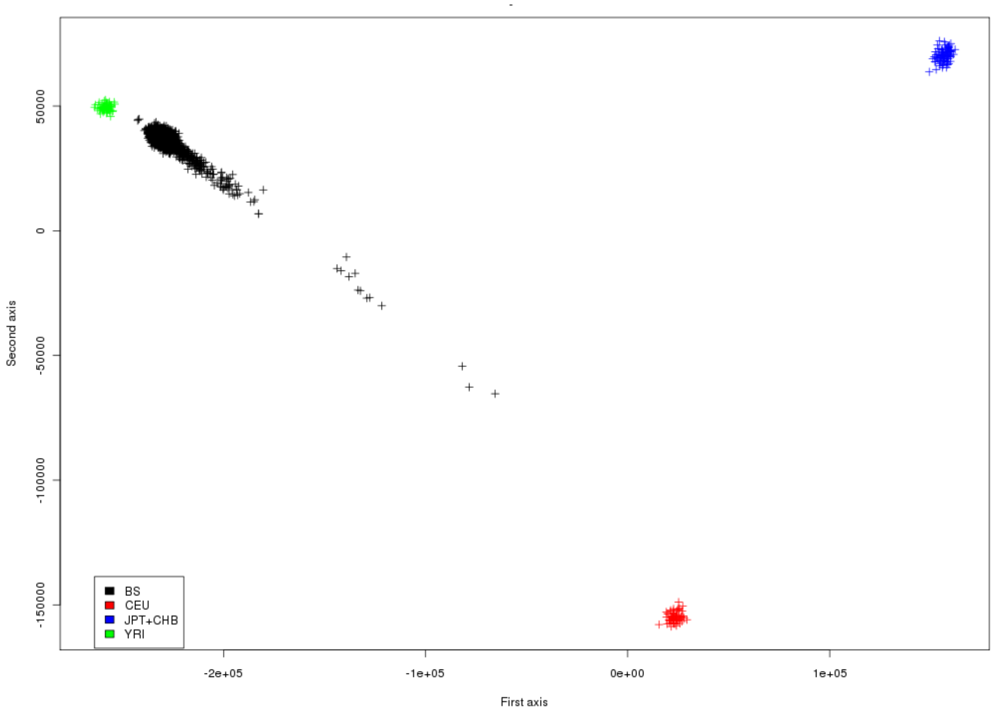

B)

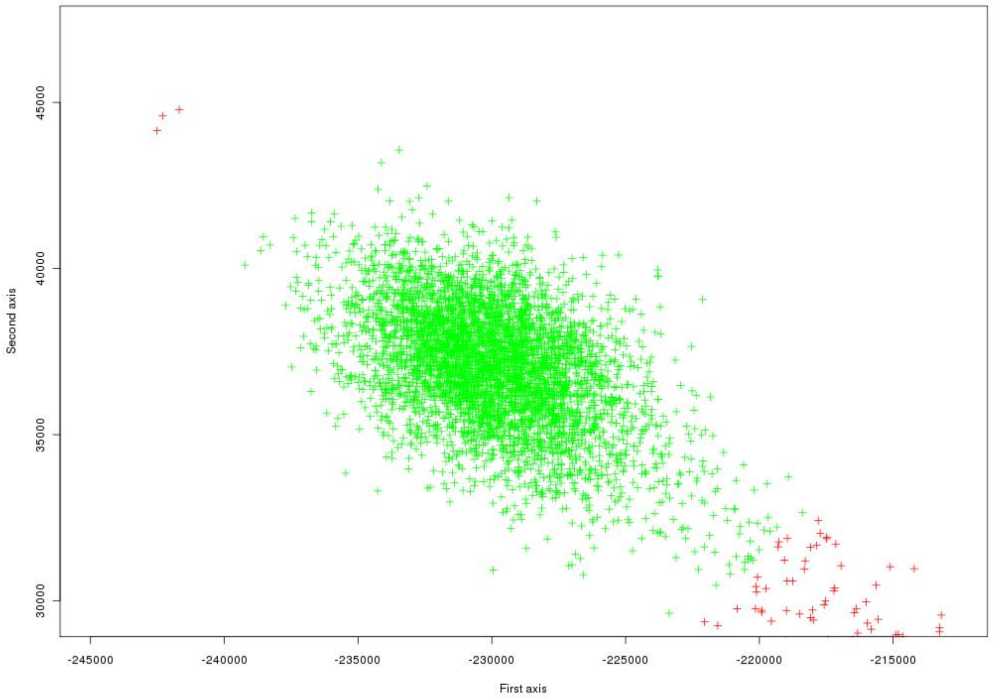

Figure S4. Relatedness shown by estimated identity by descent (IBD) across the genome for the 4000 most related sample pairs. Each vertical line represents a pairwise comparison of two individuals: red indicates two alleles IBD, blue indicates one allele IBD, and grey indicates zero alleles IBD. Thereby duplicate samples (or monozygotic twins) are shown with a full red line. Sibling pairs result in lines that are approximately one quarter red, half blue, and one quarter grey. Partly grey and partly blue lines represent sample pairs that are less related, i.e. cousins, second cousins etc. The dotted horizontal lines indicate 25% centiles.

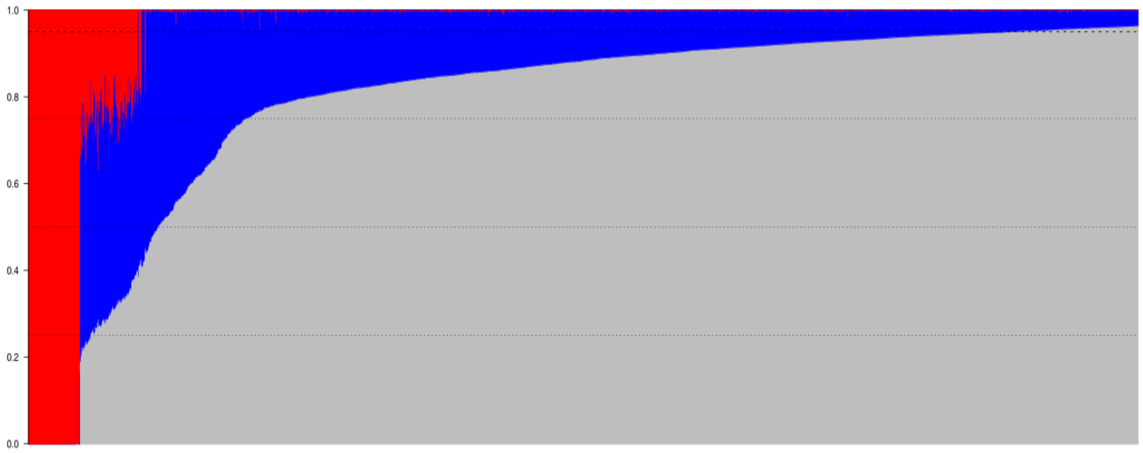

Figure S5. The first and second axes of the principal components analysis in the discovery dataset using 168,217 SNPs. Color coding represents A) reported ethnicity and B) case control status.

A)

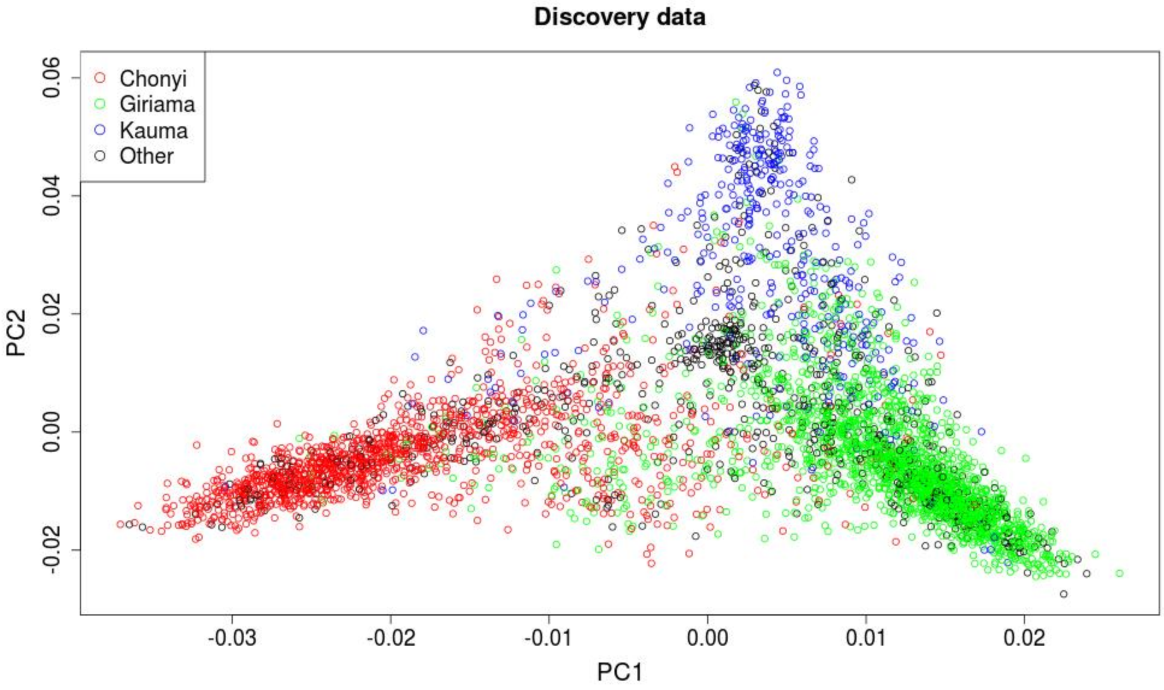

B)

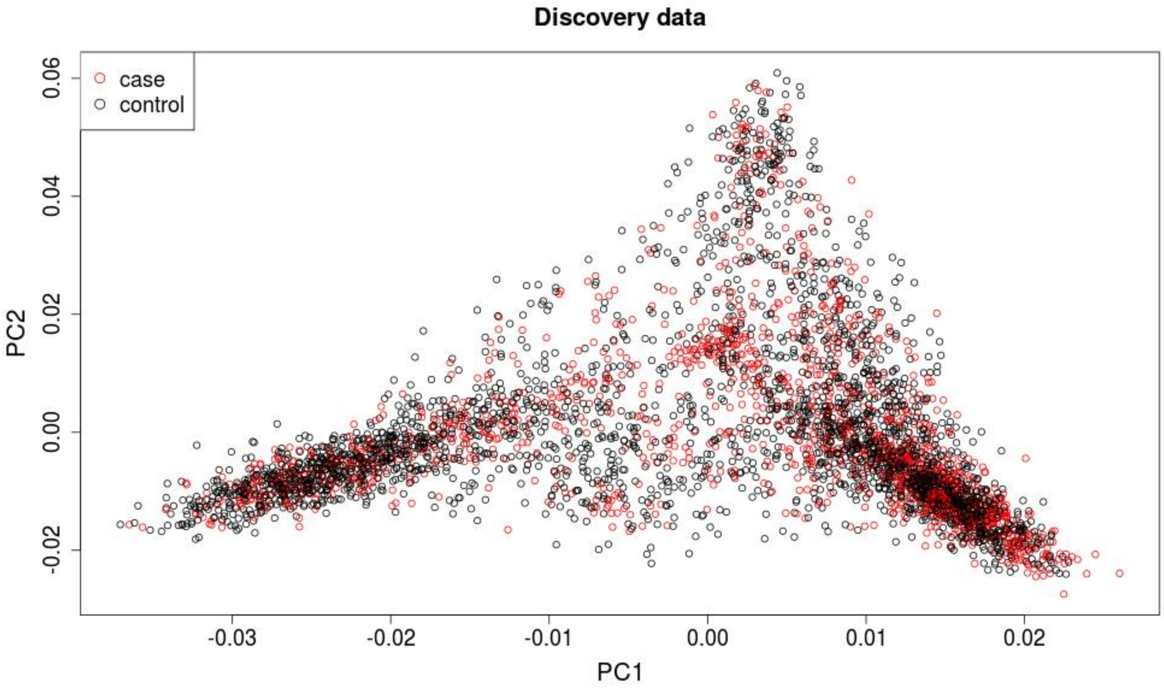

Figure S6. The first and second axes of the principal components analysis in the ImmunoChip replication dataset using 27,026 SNPs. Color coding represents A) reported ethnicity, B) case control status.

A)

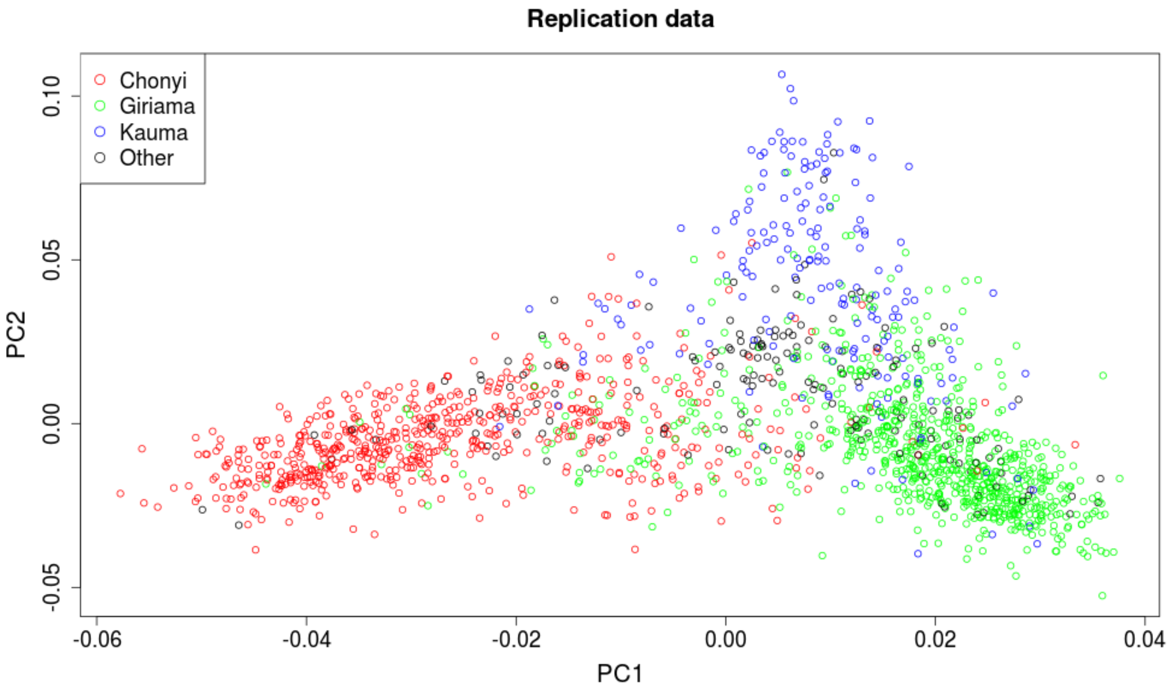

B)

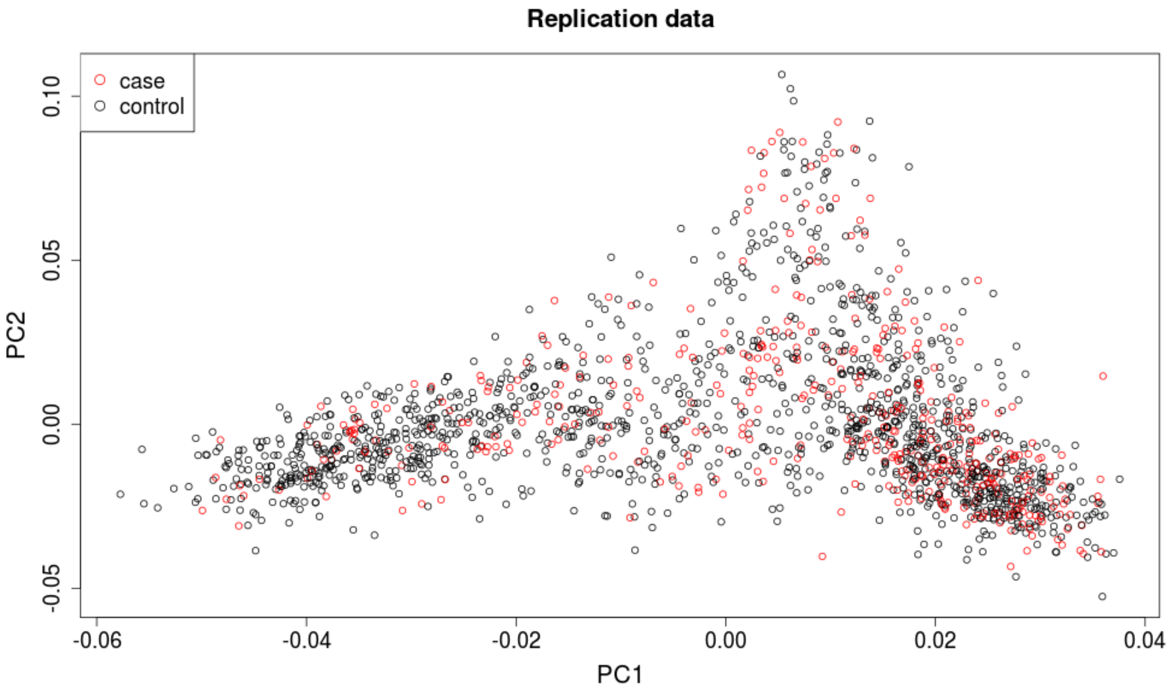

Figure S7. QQ-plots of the pneumococcal bacteremia (λ=1.013) and bacteremia overall (λ=1.043) analyses after imputation and data cleaning. Association analyses were performed with SNPTEST2 (additive model). After the SNPTEST analysis, every SNP with a minor allele frequency < 0.02, info < 0.8, or Hardy-Weinberg equilibrium P < $1x10^{-10}$ were excluded.

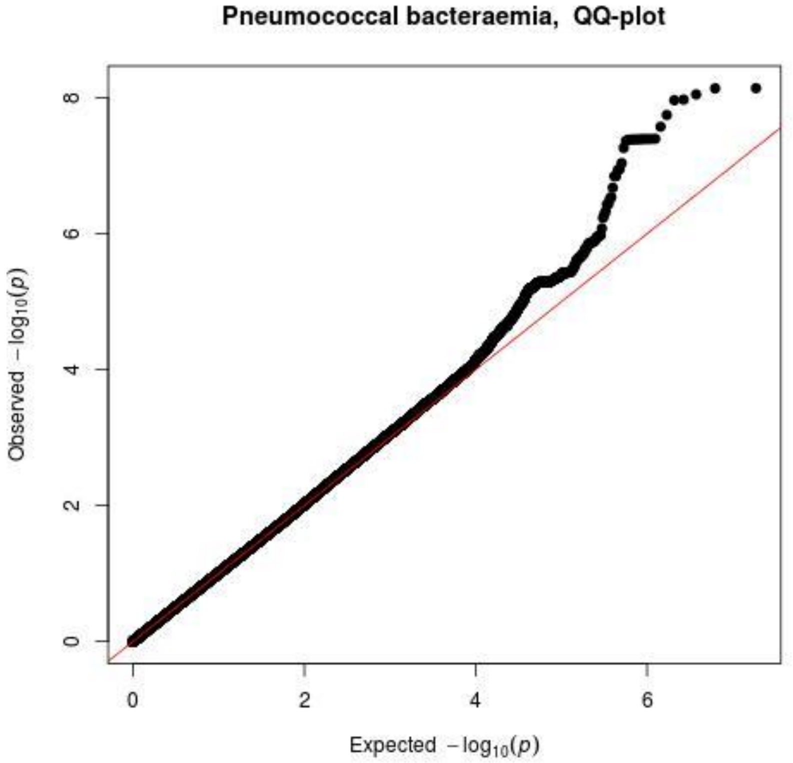

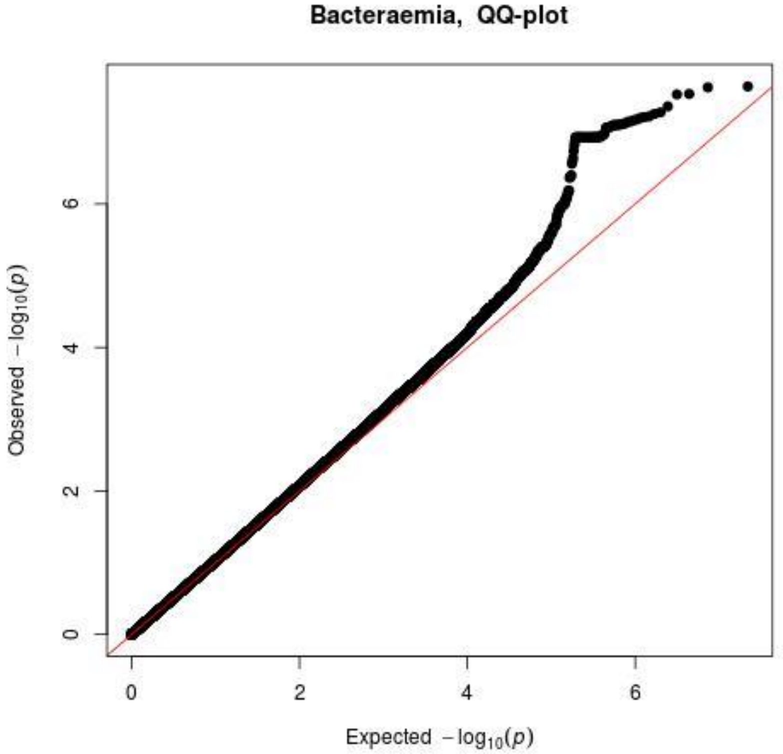

Figure S8. Sequenom cluster plot for the rs140817150 SNP (chr7 lincRNA, pneumococcal bacteremia).

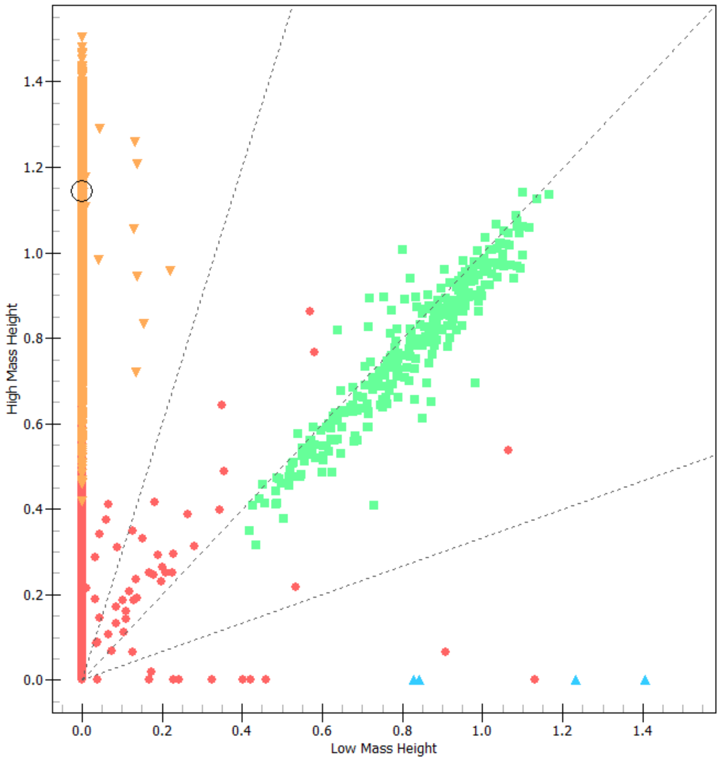

Figure S9. Statistical power to detect an association with a p-value of 0.05 in the replication data set (434 cases and 1336 controls) with varying odds ratio and minor allele frequency (MAF) as indicated.

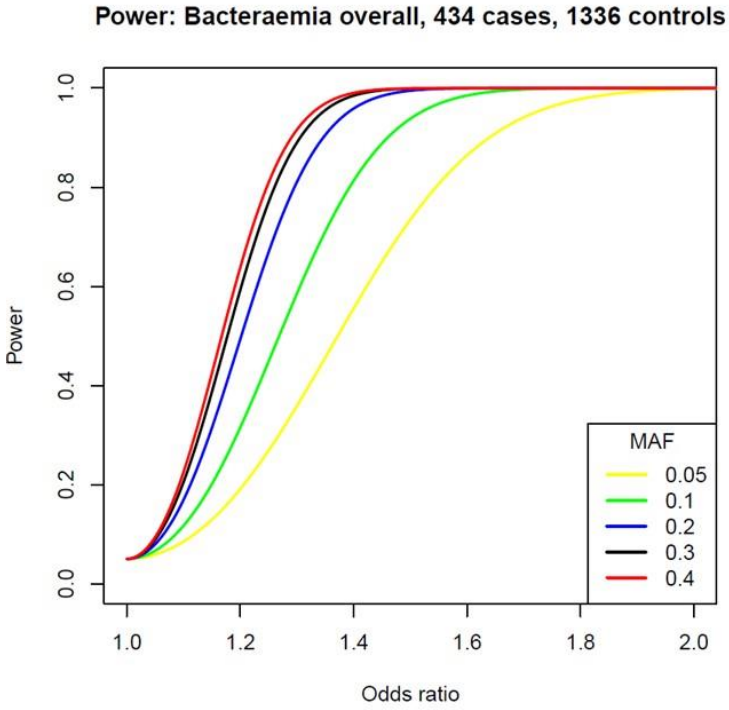

Figure S10. Manhattan plot for the pneumococcal bacteremia discovery analysis (additive model) after imputation. SNPs with minor allele frequency > 2% and info value > 0.9 are included (9,337,574 SNPs).

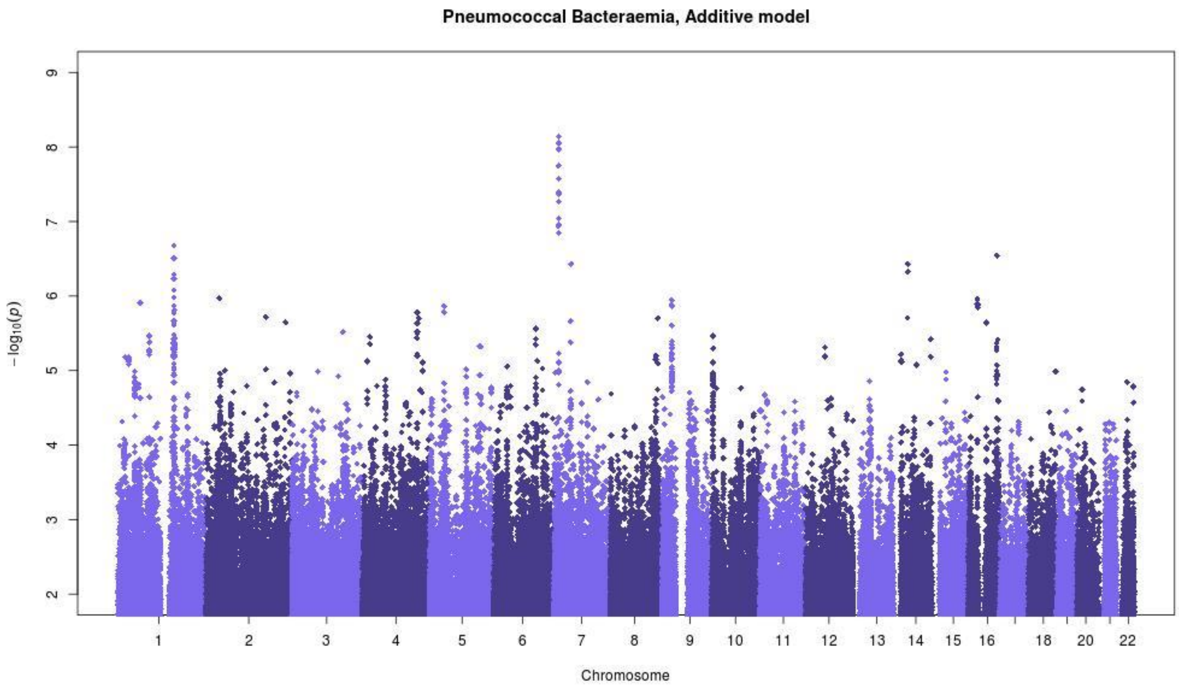

Figure S11. rs140817150 lincRNA and rs334 *HBB* associations with pneumococcal bacteremia and bacteremia overall, respectively, in the discovery data set stratified by the main ethnic groups.

A) Left panel: The three largest ethnic groups with the first two principal components of genetic structure. Right panel: Manual clustering of the individuals into four groups reflecting reported ethnicities. Sizes of groups are in the legend.

B) Log odds-ratios separately for the four groups defined in the right panel of (A), and their combined estimate.

A)

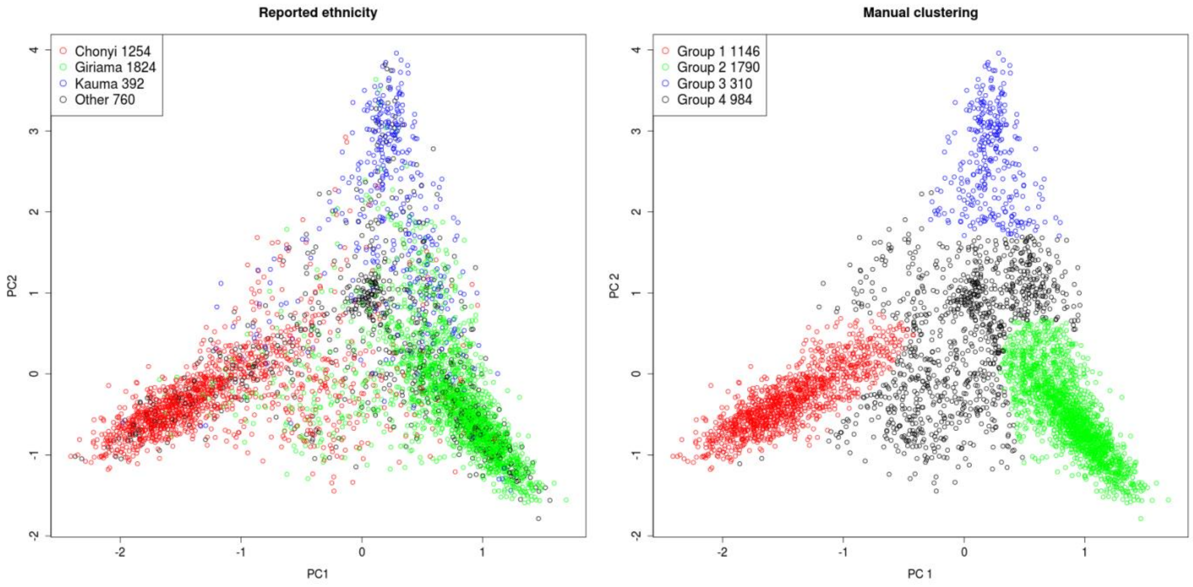

B)

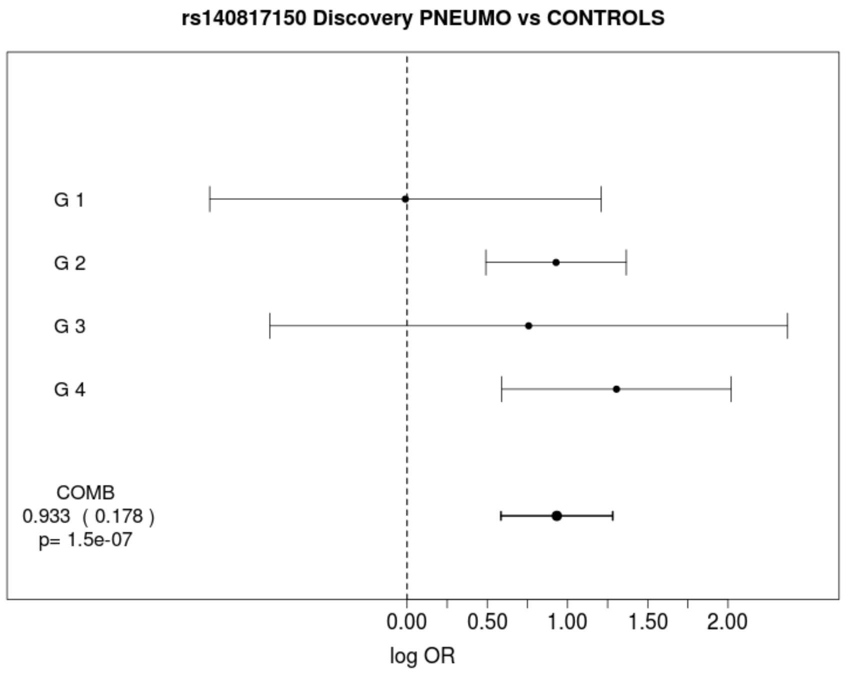

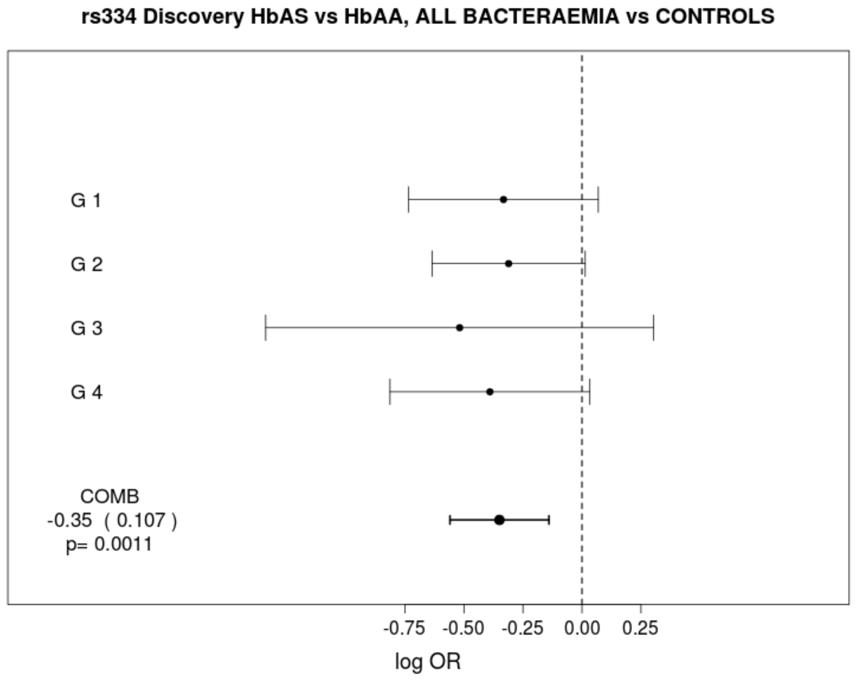
rs334 Discovery HbAS vs HbAA, ALL BACTERAEMIA vs CONTROLS
G 1
G 2
G 3
G 4
COMB
-0.35 (0.107)
p= 0.0011
-0.75 -0.50 -0.25 0.00 0.25
log OR

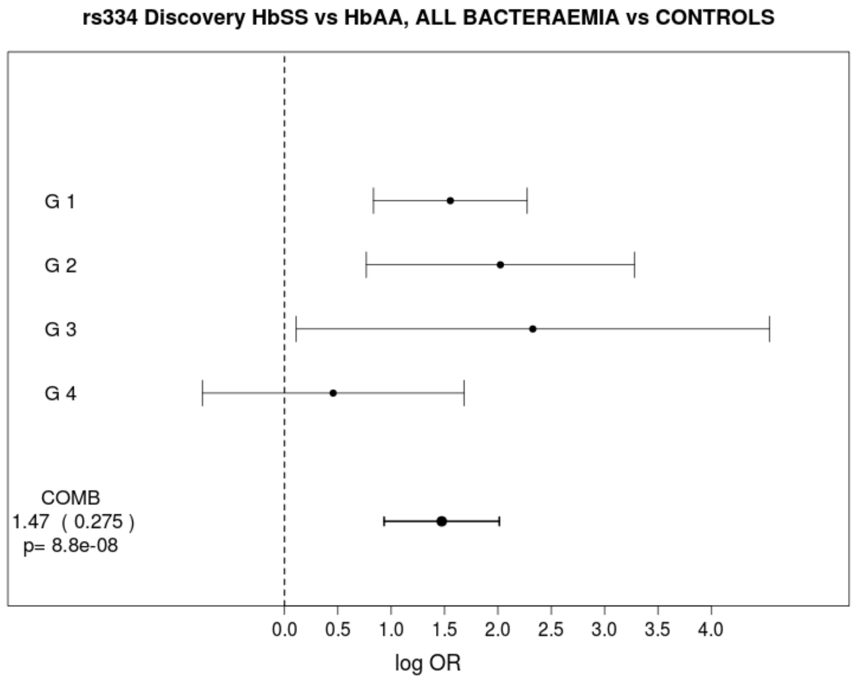
rs334 Discovery HbSS vs HbAA, ALL BACTERAEMIA vs CONTROLS
G 1
G 2
G 3
G 4
COMB
1.47 (0.275)
p= 8.8e-08
0.0 0.5 1.0 1.5 2.0 2.5 3.0 3.5 4.0
log OR

Figure S12. Manhattan plots for the bacteremia overall discovery analysis after imputation in the additive (A) and the genotypic (B) models. SNPs with minor allele frequency of > 2% and a model specific info value > 0.8 are included (10,996,498 and 8,156,976 SNPs in the additive and genotypic models respectively).

A)

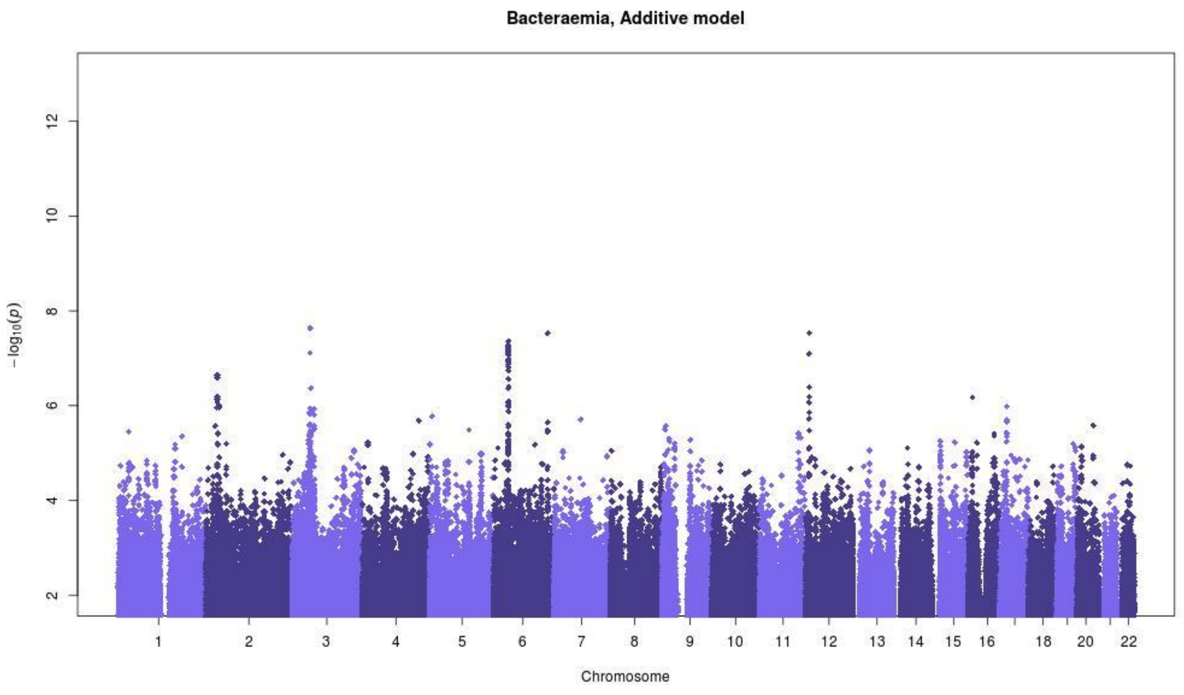

B)

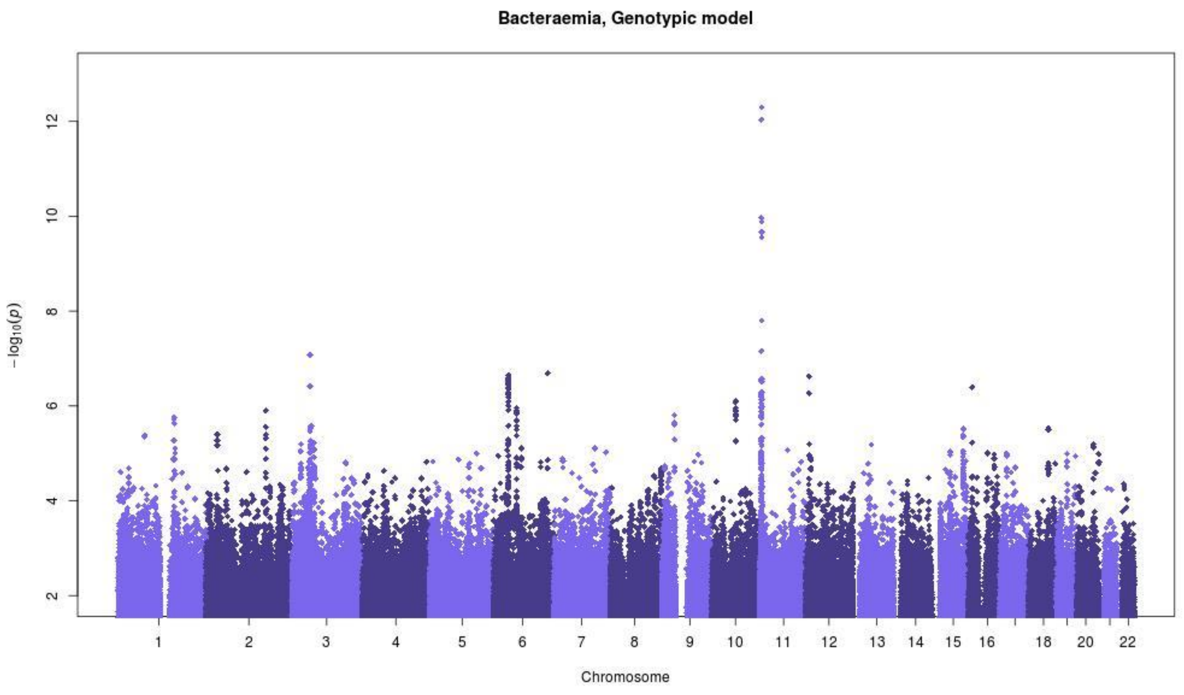

Figure S13. *HBB* association (rs334) with the main bacterial infections. Left panel: Log transformed combined odds ratios and 95% confidence intervals of directly genotyped discovery and replication samples. The dotted line represents the log OR of 0 (OR of 1; no difference between cases and controls). The values of point estimates and standard errors (in parentheses) are also given. Bacterial infections: PNEUM=*Streptococcus pneumoniae* (pneumococcus); ACINET=*Acinetobacter* species; HAEMOPH= *Haemophilus influenzae*; ECOLI=*Escherichia coli*; SALMON= *Salmonella* (non-typhoidal); STREPBH=*Streptococcus beta hemolytic*; SAUR=*Staphylococcus aureus*. Right panel: The posterior probabilities on the models of association: no effect in any subtype (NULL), same effect in all subtypes (SAME), related effects across subtypes (REL) or a same non-zero effect only in ECOLI (E) (See Methods). Models are a priori assumed to be equally likely. Bayes factors, which compare the evidence (marginal likelihood) between any pair of models, can be calculated as the ratio of the posterior probability assigned to each model as reported under each bar of the plot.

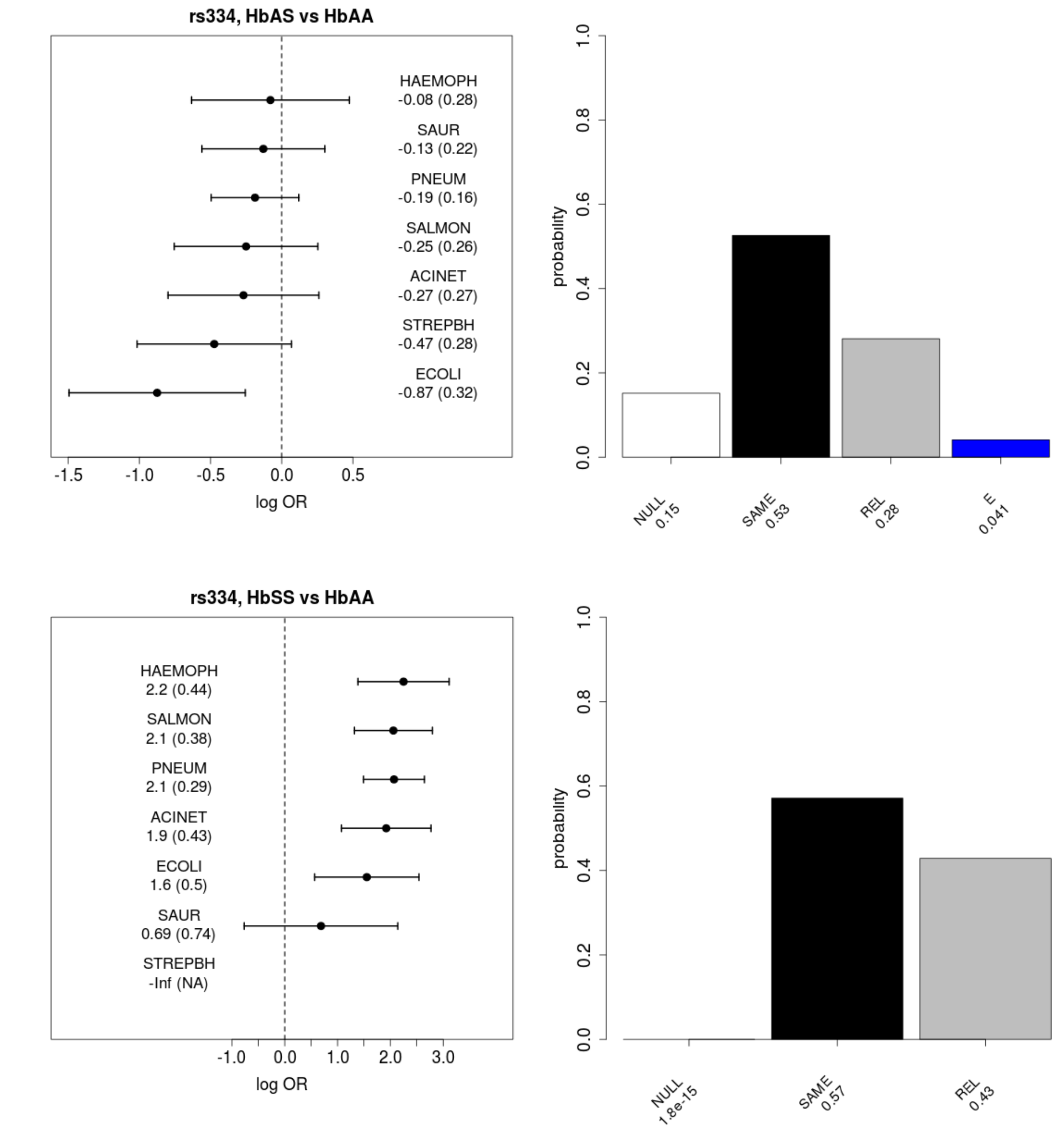

Supplemental Tables

Table S1. Sample exclusions in the discovery data and ImmunoChip replication sets.

**Exclusion criteria**	**Number of excluded discovery samples**	**Number of excluded ImmunoChip replication samples**
Call rate and extreme heterozygosity	182	84
HapMap PCA outliers	186	51
Outlying channel intensity	14	4
Sequenom disconcordance (N that otherwise passed the QC)	17 (5)	8 (7)
Discrepant or undetermined gender (N that otherwise passed the QC)	110 (32)	5 (0)
Intentional duplicate samples for QC purposes	16	0
Duplicate samples	87	6
Relatedness (r>0.4)	117	29
Duplicates when compared with discovery samples	NA	29
**Exclusions based on phenotype (altogether):**	**130**	**37**
Missing clinical data	3	0
Contaminant, not bacteremia	9	0
Fungemia, not bacteremia	4	0
Meningitis, not bacteremia	44	0
Not bacteremia	25	1
Pneumonia, empyema, not bacteremia	15	0
Uncertain pathogen	30	1
Malaria, not bacteremia	0	35
**Samples after QC**	**4213**	**1770**

Table S2. Demographic data for cases and controls in discovery and replication sets.

	Cases (discovery; N=1536)	Cases (replication; N=434)	Controls (discovery; N=2677)	Controls (replication; N=1336)
Mean age in years (range)*	2.3 (0-13)	2.1 (0-13)	5.1 (0-6.9)	4.9 (0-6.7)
Proportion of individuals aged <2 months	16.5 %	26.5 %	0.19%	0.2%
Proportion of individuals aged 2 months – 2 years	47.4 %	39.7 %	5.0%	6.4%
Proportion of individuals aged 2 – 5 years	21.2 %	22.5 %	23.8%	43.8%
Proportion of individuals more than 5 years	14.8 %	11.4 %	70.7%	49.3%
Missing age information	0	0	0.3%	0.3%
Bacteremia	100%	100%	0.3%	0.2%
Mortality	25.8 %	26.1 %	0.9%	1.9%
Females	42.7 %	47.0 %	49.6 %	49.5 %
Reported ethnicity: Giriama	57.8 %	54.3 %	45.8 %	40.0 %
Reported ethnicity: Chonyi	24.9 %	25.0 %	37.1 %	44.1 %
Reported ethnicity: Kauma	7.3 %	12.5 %	11.9 %	10.3 %

*Controls belong to a birth cohort, hence all these children were less than 12 months of age when recruited to the study. The age of controls listed in this table refers to the age at the latest follow-up date.

Table S3. Distribution of the most common bacterial species in the discovery (1536 cases) and replication sets (434 cases).

**Bacterial species**	**Discovery samples**	**Replication samples**
Streptococcus pneumoniae	429 (27.9%)	113 (26.0%)
Salmonella (non-Typhi)	180 (11.7%)	38 (8.8%)
Staphylococcus aureus	178 (11.6%)	46 (10.6%)
Escherichia coli	162 (10.6%)	43 (9.9%)
Streptococcus beta-hemolytic	160 (10.4%)	48 (11.1%)
Haemophilus influenzae	134 (8.7%)	31 (7.1%)
Acinetobacter species	130 (8.5%)	48 (11.1%)

Table S4. Association results of the bacteremia overall and pneumococcal bacteramia analyses (additive test) after direct genotyping. GWAS discovery analysis (logistic regression using the first two principal components as covariates in PLINK) included 1536 cases with microbiologically proven bacteremia (429 pneumococcal) and 2677 controls. Approximately 2000 SNPs were included in the ImmunoChip replication (approximately $P < 1\text{x}10^{-3}$), which were genotyped in 434 cases (113 pneumococcal) and 1336 controls. All of the SNPs with $P < 1\text{x}10^{-5}$ in the discovery analyses are included in the table. In addition, SNPs with $P < 10^{-3}$ are shown, if they replicated in the same direction with $P < 0.05$. SNPs highlighted in bold show suggestive association and replication in the same direction, although none of these reached the genome-wide significance level of $P < 5\text{x}10^{-8}$. OR = odds ratio; "Effects" shows the direction of association in the discovery and replication sets ("+" = minor allele confers a risk; "-" = minor allele is protective).

CHR	SNP	BP (b37)	Sub phenotype	P discovery	OR discovery	P replication	OR replication	P combined	OR combined	Effects	Gene ID
**1**	**rs6669008**	**114166561**	**overall**	**0.000884**	**1.27**	**0.00078**	**1.48**	**4.71E-06**	**1.32**	**++**	***MAGI3***
2	rs207413	31539280	overall	1.32E-06	1.58	0.9735	0.99	1.76E-05	1.44	+-	
**2**	**rs6716368**	**139924118**	**overall**	**0.000127**	**1.26**	**0.02504**	**1.27**	**9.24E-06**	**1.26**	**++**	
**3**	**rs10510428**	**14617925**	**overall**	**0.000253**	**1.18**	**0.02187**	**1.2**	**1.57E-05**	**1.19**	**++**	
**3**	**rs3922843**	**38624343**	**overall**	**0.000113**	**0.81**	**0.04668**	**0.83**	**1.47E-05**	**0.82**	**--**	***SCN5A***
3	rs9869826	50998816	overall	2.88E-08	0.71	0.7553	1.03	4.43E-06	0.79	-+	*DOCK3*
**3**	**rs7633054**	**62138954**	**overall**	**8.23E-07**	**1.39**	**0.04803**	**1.27**	**1.45E-07**	**1.36**	**++**	***PTPRG***
**3**	**rs9853409**	**62157762**	**overall**	**3.67E-06**	**1.34**	**0.3831**	**1.10**	**8.40E-06**	**1.28**	**++**	***PTPRG***
**4**	**rs4694744**	**70326656**	**overall**	**2.98E-05**	**0.65**	**0.00635**	**0.57**	**7.22E-07**	**0.63**	**--**	
**4**	**rs1080755**	**70344641**	**overall**	**0.000203**	**0.68**	**0.0265**	**0.65**	**1.57E-05**	**0.68**	**--**	***UGT2B4***
**4**	**rs13116232**	**70358525**	**overall**	**0.000219**	**0.68**	**0.0145**	**0.60**	**1.12E-05**	**0.67**	**--**	***UGT2B4***
**4**	**rs1500801**	**169668377**	**overall**	**0.000885**	**1.52**	**0.00068**	**1.99**	**3.96E-06**	**1.64**	**++**	***PALLD***
**5**	**rs11959975**	**9902829**	**overall**	**0.000229**	**1.25**	**0.0067**	**1.33**	**5.32E-06**	**1.27**	**++**	***LOC285692***
**5**	**rs7700462**	**74332599**	**overall**	**0.000224**	**1.26**	**0.0234**	**1.29**	**1.55E-05**	**1.27**	**++**	***GCNT4***
**5**	**rs7725038**	**145388180**	**overall**	**9.91E-06**	**1.34**	**0.1132**	**1.20**	**3.98E-06**	**1.3**	**++**	***SH3RF2***
**5**	**rs9325001**	**145393315**	**overall**	**9.00E-06**	**1.34**	**0.2409**	**1.14**	**1.06E-05**	**1.29**	**++**	***SH3RF2***
6	rs12181583	42154609	overall	6.10E-06	0.8	0.5767	0.95	2.79E-05	0.83	--	
6	rs9369346	42159132	overall	1.13E-07	0.75	0.2948	1.10	5.67E-05	0.83	-+	*GUCA1B*
6	rs13217993	42164401	overall	5.33E-06	0.76	0.2594	1.12	0.000858	0.84	-+	*GUCA1B*
6	rs6912658	42167161	overall	4.39E-07	0.77	NA	NA	NA	NA	NA	*GUCA1B*
6	rs4424082	42194235	overall	3.32E-07	0.76	0.01507	1.26	0.001433	0.86	-+	*MRPS10*
**6**	**rs1871693**	**136475578**	**overall**	**0.000313**	**0.79**	**0.04618**	**0.80**	**3.85E-05**	**0.79**	**--**	***PDE7B***
6	rs4286796	152681049	overall	3.44E-06	1.30	0.9791	1.00	5.43E-05	1.22	+-	*SYNE1*
7	rs6961481	25256245	overall	9.84E-06	0.79	0.1371	1.14	0.002531	0.87	-+	*NPVF*
**11**	**rs800160**	**2380134**	**overall**	**7.90E-05**	**1.23**	**0.00695**	**1.27**	**1.91E-06**	**1.24**	**++**	
**11**	**rs10840270**	**9629553**	**overall**	**3.34E-05**	**0.80**	**0.02075**	**0.80**	**2.04E-06**	**0.8**	**--**	
11	rs17185574	61745694	overall	9.34E-06	0.39	0.3221	1.34	0.002646	0.59	-+	
**12**	**rs9652020**	**40547379**	**overall**	**0.000212**	**0.80**	**0.01168**	**0.77**	**7.90E-06**	**0.79**	**--**	
**12**	**rs2873193**	**133484722**	**overall**	**0.000478**	**1.21**	**0.03069**	**1.23**	**3.79E-05**	**1.22**	**++**	***CHFR***
17	rs11655073	31736656	overall	6.53E-06	2.02	NA	NA	NA	NA	NA	*ACCN1*
**18**	**rs2111287**	**54933562**	**overall**	**0.000626**	**0.86**	**0.04462**	**1.18**	**7.23E-05**	**0.85**	**--**	

**18**	**rs7240462**	**69662581**	**overall**	**4.35E-05**	**0.83**	**0.06037**	**0.86**	**7.51E-06**	**0.84**	**--**	
1	rs6669271	156738623	pneumococcal	1.05E-06	1.50	0.6652	0.93	2.73E-05	1.37	+-	
1	rs11264997	156752106	pneumococcal	3.81E-06	1.47	0.7004	0.94	7.57E-05	1.34	+-	
1	rs12145401	156761317	pneumococcal	6.03E-06	1.46	0.9775	1.00	5.08E-05	1.35	+-	
1	rs2054991	156772707	pneumococcal	5.06E-06	1.46	0.6475	0.93	0.000102	1.34	+-	
1	rs10908677	156772810	pneumococcal	2.30E-06	1.46	0.6475	0.93	0.000049	1.34	+-	
1	rs923663	156774356	pneumococcal	8.37E-06	1.45	0.6475	0.93	0.00015	1.33	+-	*OR6Y1*
1	rs863345	156801606	pneumococcal	6.17E-07	1.63	NA	NA	NA	NA	NA	
**3**	**rs3913941**	**21468206**	**pneumococcal**	**0.00036**	**1.39**	**0.03406**	**1.45**	**0.000036**	**1.4**	**++**	***ZNF659***
**3**	**rs4858325**	**21468776**	**pneumococcal**	**0.00057**	**1.37**	**0.04191**	**1.43**	**6.44E-05**	**1.39**	**++**	***ZNF659***
4	rs7661138	156124175	pneumococcal	3.30E-06	1.55	0.01081	0.54	0.000661	1.35	+-	
5	rs6452345	26604000	pneumococcal	6.73E-07	3.70	0.3556	0.39	4.69E-06	3.22	+-	
6	rs1570187	123982387	pneumococcal	8.22E-06	0.71	0.4033	0.89	1.59E-05	0.75	--	*TRDN*
7	rs2723392	13395217	pneumococcal	0.00094	1.40	0.01648	1.57	5.14E-05	1.44	++	
7	rs1347077	13396229	pneumococcal	0.00093	1.40	0.01648	1.57	5.14E-05	1.44	++	
**7**	**rs17155006**	**107545079**	**pneumococcal**	**0.00017**	**0.75**	**0.01443**	**0.70**	**7.90E-06**	**0.74**	**--**	***LAMB4***
8	rs3739262	134274895	pneumococcal	2.51E-06	1.63	0.4737	1.17	5.23E-06	1.53	++	*WISP1*
**13**	**rs9526114**	**45306038**	**pneumococcal**	**4.93E-05**	**1.35**	**0.04376**	**1.33**	**5.83E-06**	**1.35**	**++**	***LOC283514***
14	rs10483548	38386980	pneumococcal	2.27E-06	1.45	0.5388	0.91	7.56E-05	1.32	+-	

Table S5. The most significant associations (additive model) in the pneumococcal bacteremia discovery analysis after imputation. Imputed discovery analysis (logistic regression using the first two principal components as covariates in SNPTEST) included 429 cases with microbiologically proven pneumococcal bacteremia and 2677 controls. All the loci with *P* < 1×10^{-6} in SNPTEST analysis are included in the table (Only the most significant SNP in each locus plus the ones with replication information available). In addition, SNPs with P < 1×10^{-5} are shown, if replication genotyping was available. SNPs highlighted in bold show suggestive association and replication. Replication genotyping was performed using the Sequenom iPLEX platform (103 cases and 1333 controls), unless denoted with a * in which case the ImmunoChip was used (113 cases and 1336 controls). OR = odds ratio; "Effects" shows the direction of association in the discovery and replication sets ("+" = minor allele confers a risk; "-" = minor allele is protective).

Directly genotyped	CHR	BP (b37)	SNP	GENE	P discovery imputed	OR discovery imputed	MAF imputed cases	MAF imputed controls	MAF genotyped cases discovery	MAF genotyped controls discovery	P genotyped discovery	OR genotyped discovery	P Replication	OR Replication	P Combined	OR Combined	Effects
**---**	**1**	**62932815**	**rs1168000**	***DOCK7***	**3.81E-06**	**2.53**	**0.0396**	**0.0183**	**0.0287**	**0.0112**	**3.686E-05**	**2.81**	**0.04553**	**3.10**	**4.64E-06**	**2.85**	**++**
**---**	**1**	**62953183**	**rs1168025**	***DOCK7***	**3.98E-07**	**3.07**	**0.0329**	**0.0123**	**0.0276**	**0.0112**	**9.211E-05**	**2.70**	**0.05296**	**2.97**	**1.32E-05**	**2.74**	**++**
---	1	158464990	rs11264984	-	3.80E-06	1.41	0.5072	0.4253	0.5266	0.4405	2.851E-06	1.42	0.8489	0.97	4.55E-05	1.31	+-
YES	1	158471999	rs6669271	-	8.37E-07	1.50	0.2867	0.2092					*0.6652	*0.93	2.73E-05	1.37	+-
---	1	158473725	rs6663286	-	2.15E-06	1.45	0.3439	0.2654	0.3338	0.2525	1.996E-06	1.49	0.3933	0.87	9.55E-05	1.34	+-
YES	1	158485482	rs11264997	-	3.29E-06	1.47	0.2949	0.2219					*0.7004	*0.94	7.57E-05	1.34	+-
YES	1	158494693	rs12145401	-	5.17E-06	1.45	0.2937	0.2223					*0.9775	*1.00	5.08E-05	1.35	+-
YES	1	158506083	rs2054991	-	6.30E-06	1.45	0.2937	0.2228					*0.6475	*0.93	0.000102	1.34	+-
YES	1	158506186	rs10908677	-	2.47E-06	1.46	0.3019	0.2260					*0.6475	*0.93	0.000049	1.34	+-
YES	1	158507732	rs923663	*OR6Y1*	6.30E-06	1.45	0.2937	0.2228					*0.6475	*0.93	0.00015	1.33	+-
YES	1	158518939	rs9804152	*OR6Y1*	9.00E-06	1.57	0.1539	0.0996					*0.6851	*1.09	4.48E-05	1.45	++
---	1	158528118	rs10752626	*OR6P1*	3.11E-07	1.64	0.1906	0.1231	0.1916	0.1216	2.725E-07	1.65	0.6004	1.11	1.17E-06	1.54	++
YES	1	158534982	rs863345	*OR6P1*	2.10E-07	1.65	0.1946	0.1253	0.1937	0.1241	3.766E-07	1.65	0.506	1.15	1.14E-06	1.55	++
**---**	**3**	**128701130**	**rs6439153**	***KIAA1257***	**4.71E-07**	**3.72**	**0.0251**	**0.0077**	**0.0413**	**0.0225**	**0.0001585**	**2.18**	**0.000608**	**3.02**	**4.99E-07**	**2.39**	**++**
YES	4	155904725	rs7661138	-	3.02E-06	1.54	0.2133	0.1526					*0.01081	*0.54	0.000661	1.35	+-
YES	5	26568243	rs6452345	-	8.26E-08	3.70	0.0280	0.0075					*0.3556	*0.39	4.69E-06	3.22	+-
---	5	169453092	rs114183953	*DOCK2*	1.95E-07	3.15	0.0335	0.0111	0.0337	0.0108	8.934E-07	3.19	0.562	1.36	2.34E-06	2.77	++
---	5	169460440	rs138715413	*DOCK2*	1.81E-07	3.16	0.0334	0.0109	0.0323	0.0110	3.778E-06	3.00	0.5461	1.38	8.06E-06	2.63	++
YES	6	38520864	rs724242	*BTBD9*	8.76E-06	0.64	0.1539	0.2185					*0.91	*0.98	0.000121	0.72	--
YES	6	123940688	rs1570187	*TRDN*	7.49E-06	0.71	0.4114	0.4836					*0.4033	*0.89	1.59E-05	0.75	--
**---**	**7**	**13394635**	**rs188755755**	**-**	**8.91E-09**	**2.72**	**0.0593**	**0.0220**	**0.0544**	**0.0214**	**1.039E-06**	**2.50**	**0.01096**	**2.40**	**3.73E-08**	**2.47**	**++**
**---**	**7**	**13396630**	**rs140817150**	**-**	**7.25E-09**	**2.74**	**0.0592**	**0.0217**	**0.0639**	**0.0266**	**3.585E-07**	**2.39**	**0.001161**	**2.72**	**1.69E-09**	**2.47**	**++**
---	7	13401014	rs116432683	-	7.20E-09	2.74	0.0592	0.0217									
---	8	134193074	rs11782936	-	5.80E-07	1.65	0.2035	0.1404									
YES	8	134205713	rs3739262	*WISP1*	1.98E-06	1.63	0.1667	0.1087					*0.4737	*1.17	5.23E-06	1.53	++
YES	9	27773059	rs775397	-	8.98E-06	1.46	0.2622	0.1946					*0.8725	*1.03	0.000057	1.36	++
---	14	39314125	rs7142347	-	3.69E-07	1.74	0.1475	0.0963									
---	14	39315768	rs73277180	-	4.71E-07	1.73	0.1470	0.0961	0.1396	0.0966	6.451E-05	1.56	0.9459	1.02	0.000224	1.46	++
YES	14	39317229	rs10483548	-	1.96E-06	1.45	0.3835	0.3106					*0.5388	*0.91	7.56E-05	1.32	+-

Table S6. The most significant associations in the chr7 lincRNA region in the pneumococcal bacteremia discovery analysis (after Affymetrix SNP 6.0 genotyping and imputation). Directly genotyped SNPs with P-values less than 0.05 and imputed SNPs with P-value less than $1x10^{-6}$ are shown in the region (250 kb region flanking the top SNP rs140817150 is shown). * denotes imputed SNPs that were confirmed by direct genotyping using Sequenom.

SNP	BP (b37) in chr 7	genotyped(G)/ imputed (I)	P discovery	OR discovery	maf cases	maf controls	Position relative to AC011288.2-001	Position relative to AC006000.5
rs6973224	13266540	G	0.043	1.17	0.371	0.340	intron 2-3	3' downstream
rs17166908	13270959	G	0.0037	0.78	0.223	0.273	intron 2-3	3' downstream
rs10230302	13275542	G	0.0016	1.33	0.220	0.170	intron 2-3	3' downstream
rs183536610	13350258	I	2.05E-07	2.42	0.061	0.027	intron 3-4	3' downstream
rs114563489	13351620	I	1.76E-07	2.44	0.061	0.026	intron 3-4	3' downstream
rs114237419	13352333	I	1.62E-07	2.44	0.061	0.026	intron 3-4	3' downstream
rs184608123	13355876	I	1.15E-07	2.48	0.061	0.026	intron 3-4	3' downstream
rs112384903	13356316	I	1.12E-07	2.48	0.061	0.026	intron 3-4	3' downstream
rs75811201	13360996	I	9.13E-08	2.50	0.061	0.025	intron 3-4	3' downstream
rs75208382	13363234	I	1.42E-07	2.51	0.059	0.024	intron 3-4	3' downstream
rs192359699	13365219	I	5.45E-08	2.55	0.061	0.025	intron 3-4	3' downstream
rs184746152	13371007	I	4.31E-08	2.57	0.060	0.024	intron 3-4	3' downstream
rs75091393	13373716	I	4.14E-08	2.58	0.060	0.024	intron 3-4	3' downstream
rs139819665	13374686	I	4.11E-08	2.58	0.060	0.024	intron 3-4	3' downstream
rs186811037	13377949	I	4.10E-08	2.58	0.060	0.024	intron 3-4	3' downstream
rs182261263	13378675	I	4.08E-08	2.58	0.060	0.024	intron 3-4	3' downstream
rs146813781	13380872	I	4.07E-08	2.58	0.060	0.024	intron 3-4	intron 3-4
rs182683147	13385227	I	4.04E-08	2.58	0.060	0.024	intron 3-4	intron 3-4
rs74757989	13386451	I	4.04E-08	2.58	0.060	0.024	intron 3-4	intron 3-4
rs78571337	13387037	I	4.03E-08	2.58	0.060	0.024	intron 3-4	intron 3-4
rs186096483	13387766	I	4.01E-08	2.58	0.060	0.024	intron 3-4	intron 3-4
rs116118621	13391291	I	1.08E-08	2.70	0.059	0.022	intron 3-4	intron 3-4
rs188755755*	13394635	I	8.91E-09	2.72	0.059	0.022	intron 3-4	intron 3-4
rs140817150*	13396630	I	7.25E-09	2.74	0.059	0.022	intron 3-4	intron 3-4
rs116432683	13401014	I	7.20E-09	2.74	0.059	0.022	intron 3-4	intron 3-4
rs114325568	13423766	I	1.43E-07	2.44	0.062	0.026	intron 3-4	5' upstream
rs2568600	13424671	G	0.0012	1.39	0.171	0.131	intron 3-4	5' upstream
rs2723392	13428692	G	0.00097	1.40	0.175	0.133	intron 3-4	5' upstream
rs1347077	13429704	G	0.00097	1.40	0.175	0.133	intron 3-4	5' upstream
rs77922834	13432361	I	1.06E-08	2.70	0.061	0.023	intron 3-4	5' upstream
rs192669340	13471405	I	2.66E-08	2.49	0.068	0.029	intron 3-4	5' upstream
rs184390046	13476586	I	1.79E-08	2.70	0.058	0.023	intron 3-4	5' upstream
rs10807764	13478678	G	0.0061	1.28	0.223	0.177	intron 3-4	5' upstream
rs17167126	13480033	G	0.00014	1.43	0.193	0.139	intron 3-4	5' upstream
rs2217612	13483120	G	0.0029	1.35	0.172	0.129	intron 3-4	5' upstream
rs17167140	13484133	G	0.001827	1.38	0.171	0.127	intron 3-4	5' upstream
rs17167142	13484328	G	0.001568	1.38	0.171	0.127	intron 3-4	5' upstream
rs1432488	13484508	G	0.00209	1.37	0.171	0.128	intron 3-4	5' upstream
rs10247084	13485416	G	0.003145	1.33	0.185	0.143	intron 3-4	5' upstream
rs1432492	13501571	G	0.015065	1.23	0.256	0.213	intron 3-4	5' upstream
rs12699509	13561595	G	0.013637	1.34	0.114	0.090	intron 3-4	5' upstream

Table S7. Comparison of association statistics after imputation and direct genotyping of discovery and replication samples. As described in the Methods section, only siblings (and more related individuals) were excluded from the main analysis. Association statistics of the following analyses are shown: the imputed discovery main analysis (SNPTEST2; frequentist method score, the first two principal components as covariates), directly genotyped discovery and replication analyses (PLINK, 2 PCA components as covariates), directly genotyped discovery and replication analyses when a mixed model (MM) was used to better account for relatedness and underlying population structure (with siblings and more distant relatives included and with all second degree and more closely related individuals (r>0.2) excluded from the analysis). Number of bacteremia cases overall (cases B), pneumococcal cases (cases P), and controls included in each analysis are indicated in each column. The chromosome 7 lincRNA hit in the pneumococcal subgroup (SNPs rs188755755 and rs140817150) reached genome-wide significance in the additive model in all of these analyses. MM =mixed model; B = bacteremia overall; P = pneumococcal bacteremia.

CHR	BP (b37)	SNP	Phenotype	Discovery (Imputed) cases B: 1536 cases P: 429 controls: 2677		Discovery (Genotyped) cases B: 1514 cases P: 418 controls: 2642		Replication (Genotyped) cases B: 407 cases P: 103 controls: 1333		Combined (Genotyped) cases B: 1921 cases P: 521 controls: 3975		MM Discovery (Genotyped; siblings included) cases B: 1519 cases P: 420 controls: 2688		MM Replication (Genotyped; siblings included) cases B: 408 cases P: 103 controls:1360		MM Combined (Genotyped; siblings included) cases B: 1927 cases P: 523 controls: 4048		MM Discovery (Genotyped; all related (r>0.2) removed) cases B: 1476 cases P: 408 controls:2543		MM Replication (Genotyped; all related (r>0.2) removed) cases B: 404 cases P: 102 controls:131 1		MM Combined (Genotyped; all related (r>0.2) removed) cases B: 1880 cases P: 510 controls: 3854	
				P	OR	P	OR	P	OR	P	OR	P	OR	P	OR	P	OR	P	OR	P	OR	P	OR
3	51009435	rs9879725	B	2.4E-08	0.71	6.9E-08	0.71	0.5085	1.07	1.6E-05	0.79	8.0E-08	0.72	0.3019	1.11	4.0E-05	0.80	0.02839	0.80	0.947	1.01	0.0564	0.84
3	51390967	rs13353505	B	3.8E-06	0.64	3.2E-05	0.67	0.9783	1.00	0.0004	0.75	8.4E-05	0.70	0.8907	1.02	0.00095	0.77	0.00832	0.66	0.891	1.04	0.0272	0.74
6	42162388	rs3749921	B	7.9E-08	0.74	2.3E-07	0.75	0.1653	1.14	0.0002	0.83	4.1E-07	0.76	0.1525	1.14	0.00028	0.84	0.01349	0.80	0.414	1.15	0.0771	0.87
6	42181960	rs1884318	B	4.4E-08	0.74	3.9E-07	0.75	0.1632	1.14	0.0002	0.84	7.2E-07	0.76	0.1501	1.14	0.0004	0.85	0.0155	0.80	0.412	1.15	0.0848	0.87
6	42184400	rs13197564	B	5.3E-08	0.74	1.6E-07	0.75	0.1493	1.15	0.0002	0.83	3.2E-07	0.76	0.1416	1.14	0.00025	0.84	0.01139	0.80	0.474	1.13	0.0601	0.86
6	152678110	rs6906442	B	3.6E-06	1.35	3.2E-05	1.32	0.536	1.08	8.1E-05	1.26	5.8E-05	1.30	0.6408	1.06	0.00017	1.24	0.06383	1.22	0.738	0.93	0.1268	1.16
6	152679729	rs9478326	B	2.3E-06	1.30	1.9E-05	1.27	0.8464	1.02	0.0001	1.20	6.3E-05	1.24	0.9014	1.01	0.00038	1.18	0.04085	1.20	0.587	0.91	0.1122	1.14
12	9350854	rs74500107	B	8.1E-08	1.80	3.3E-07	1.68	0.3738	0.85	4.7E-05	1.44	8.0E-07	1.63	0.3668	0.84	7.9E-05	1.41	0.0763	1.36	0.685	0.87	0.1621	1.24
12	9358781	rs149632973	B	4.1E-07	1.75	2.8E-06	1.70	0.3227	0.82	0.0003	1.43	9.9E-06	1.63	0.3286	0.82	0.0006	1.39	0.14336	1.33	0.883	0.95	0.2233	1.23
1	62932815	rs1168000	P	3.8E-06	2.53	3.7E-05	2.81	0.0455	3.10	4.6E-06	2.85	3.0E-05	2.76	0.0463	2.90	4.6E-06	2.79	8.5E-05	2.65	0.041	2.99	1.2E-05	2.70
1	62953183	rs1168025	P	4E-07	3.07	9.2E-05	2.70	0.053	2.97	1.3E-05	2.74	8.2E-05	2.64	0.0541	2.78	1.4E-05	2.66	0.00022	2.53	0.049	2.85	3.4E-05	2.58
1	158464990	rs11264984	P	3.8E-06	1.41	2.9E-06	1.42	0.8489	0.97	4.6E-05	1.31	7.2E-07	1.45	0.8720	0.98	2.1E-05	1.33	1.5E-06	1.44	0.868	0.98	3.8E-05	1.32
1	158473725	rs6663286	P	2.2E-06	1.45	2E-06	1.49	0.3933	0.87	9.6E-05	1.34	1.9E-06	1.47	0.4061	0.87	0.00011	1.33	6.8E-07	1.50	0.433	0.88	5.4E-05	1.35
1	158528118	rs10752626	P	3.1E-07	1.64	2.7E-07	1.65	0.6004	1.11	1.2E-06	1.54	7.6E-08	1.68	0.6203	1.11	6.2E-07	1.55	2.8E-07	1.66	0.627	1.10	1.9E-06	1.53
1	158534982	rs863345	P	2.1E-07	1.65	3.8E-07	1.65	0.506	1.15	1.1E-06	1.55	1.1E-07	1.68	0.5136	1.14	5.8E-07	1.56	3.7E-07	1.65	0.520	1.14	1.7E-06	1.54
5	169453092	rs114183953	P	1.9E-07	3.15	8.9E-07	3.19	0.562	1.36	2.3E-06	2.77	1.7E-07	3.39	0.6620	1.24	1.4E-06	2.82	4.1E-07	3.34	0.592	1.31	2.3E-06	2.79
5	169460440	rs138715413	P	1.8E-07	3.16	3.8E-06	3.00	0.5461	1.38	8.1E-06	2.63	1.0E-06	3.16	0.6367	1.27	5.2E-06	2.67	2.2E-06	3.11	0.569	1.33	8.6E-06	2.64
**7**	**13394635**	**rs188755755**	**P**	**8.9E-09**	**2.72**	**1E-06**	**2.50**	**0.011**	**2.40**	**3.7E-08**	**2.47**	**2.6E-07**	**2.57**	**0.0060**	**2.59**	**9.2E-09**	**2.58**	**1.7E-07**	**2.65**	**0.004**	**2.72**	**4.6E-09**	**2.67**
**7**	**13396630**	**rs140817150**	**P**	**7.3E-09**	**2.74**	**3.6E-07**	**2.39**	**0.0012**	**2.72**	**1.7E-09**	**2.47**	**9.7E-08**	**2.45**	**0.0005**	**2.96**	**5.1E-10**	**2.55**	**3.6E-08**	**2.56**	**3E-4**	**3.06**	**1.5E-10**	**2.66**
14	39315768	rs73277180	P	4.7E-07	1.73	6.5E-05	1.56	0.9459	1.02	0.0002	1.46	9.7E-05	1.54	0.9666	0.99	0.00044	1.43	0.0001	1.54	0.962	0.99	0.0005	1.43
16	26762549	rs142637346	P	1.28E-06	2.36	3.7E-06	2.37	0.5861	1.25	9.2E-06	2.13	1.8E-06	2.38	0.5383	1.29	5.3E-06	2.15	2.7E-06	2.37	0.563	1.27	8.2E-06	2.13
16	26775675	rs140127109	P	1.07E-06	2.37	4.2E-06	2.36	0.6124	1.23	1.1E-05	2.11	2.0E-06	2.37	0.5656	1.27	6.1E-06	2.14	2.8E-06	2.37	0.591	1.25	9.1E-06	2.12
16	26786471	rs148926738	P	1.11E-06	2.35	8.9E-05	2.16	0.3186	1.47	8.1E-05	2.00	6.1E-05	2.17	0.2866	1.50	5.7E-05	2.01	0.0001	2.14	0.306	1.48	9.7E-05	1.98

Table S8. The most significant associations (additive and genotypic models) in the bacteremia overall discovery analysis after imputation. Imputed discovery analysis (logistic regression using the first two principal components as covariates in SNPTEST) included 1536 cases with microbiologically proven bacteremia and 2677 controls. All the loci with p< $1x10^{-6}$ in the SNPTEST analysis are included in the table (Only the most significant SNP in the locus plus the ones with replication information available are shown). In addition, SNPs with <$1x10^{-5}$ are shown, if replication genotyping was available. SNPs highlighted in bold show suggestive association and replication. Replication genotyping was performed using the Sequenom iPlex platform (407 cases and 1333 controls), unless denoted with * in which case the ImmunoChip was used (434 cases and 1336 controls). Only the additive model is shown if both of the models passed the criteria described above. OR = odds ratio; "Effects" shows the direction of association in the discovery and replication sets ("+" = minor allele confers a risk; "-" = minor allele is protective).

Directly genotyped	CHR	BP (b37)	SNP	GENE	P discovery imputed	OR discovery imputed	MAF imputed cases	MAF imputed controls	MAF genotyped cases	MAF genotyped controls	P genotyped discovery	OR genotyped discovery	P Replication	OR Replication	P Combined	OR Combined	Effects	Model
YES	1	30169447	rs11808436		3.58E-06	1.25	0.359	0.309					*0.2624	*0.91	0.0005	1.16	+-	Additive
---	2	31300106	rs17010685	*GALNT14*	2.27E-07	2.18	0.031	0.017	0.043	0.035	0.04829	1.26	0.1479	0.70	0.25224	1.13	+-	Additive
YES	2	31539280	rs207413		1.09E-06	1.58	0.076	0.051					*0.9735	*0.99	1.8E-05	1.44	+-	Additive
YES	3	50998816	rs9869826	*DOCK3*	2.30E-08	0.71	0.150	0.202					*0.7553	*1.03	4.4E-06	0.79	-+	Additive
---	3	51009435	rs9879725	*DOCK3*	2.36E-08	0.71	0.151	0.203	0.147	0.196	6.9E-08	0.71	0.5085	1.07	1.6E-05	0.79	-+	Additive
---	3	51390967	rs13353505	*DOCK3*	3.84E-06	0.64	0.054	0.080	0.057	0.082	3.2E-05	0.67	0.9783	1.00	0.00037	0.75	-+	Additive
---	3	53783784	rs898420	*CACNA1D*	4.25E-07	1.28	0.384	0.342										Additive
**YES**	**3**	**62138954**	**rs7633054**	***PTPRG***	**1.18E-06**	**1.38**	**0.151**	**0.110**					***0.04803**	***1.27**	**1.5E-07**	**1.36**	**++**	**Additive**
YES	3	62157762	rs9853409	*PTPRG*	2.95E-06	1.34	0.174	0.135					*0.3831	*1.10	8.4E-06	1.28	++	Additive
---	3	62160653	rs17066238	*PTPRG*	2.96E-06	1.45	0.109	0.078	0.116	0.091	0.00022	1.33	0.8184	0.97	0.00186	1.23	+-	Additive
YES	6	42154609	rs12181583	*GUCA1B*	5.89E-06	1.25	0.253	0.301					*0.5767	*0.95	2.8E-05	0.83	--	Additive
YES	6	42159132	rs9369346	*GUCA1B*	1.18E-07	0.75	0.208	0.264					*0.2948	*1.10	5.7E-05	0.83	-+	Additive
---	6	42162388	rs3749921	*GUCA1B*	7.90E-08	0.74	0.201	0.257	0.202	0.257	2.3E-07	0.75	0.1653	1.14	0.00017	0.83	-+	Additive
YES	6	42164401	rs13217993	*GUCA1B*	3.92E-06	0.76	0.169	0.217					*0.2594	*1.12	0.00086	0.84	-+	Additive
---	6	42181960	rs1884318	*MRPS10*	4.35E-08	0.74	0.200	0.257	0.202	0.257	3.9E-07	0.75	0.1632	1.14	0.00024	0.84	-+	Additive
---	6	42184400	rs13197564	*MRPS10*	5.29E-08	0.74	0.207	0.264	0.208	0.264	1.6E-07	0.75	0.1493	1.15	0.00015	0.83	-+	Additive
YES	6	42194235	rs4424082	*MRPS10*	2.74E-07	0.76	0.205	0.261					*0.01507	*1.26	0.00143	0.86	-+	Additive
---	6	152672540	rs4632900	*SYNE1*	2.96E-08	1.41	0.188	0.145										Additive
---	6	152678110	rs6906442	*SYNE1*	3.57E-06	1.35	0.163	0.129	0.148	0.117	3.2E-05	1.32	0.536	1.08	8.1E-05	1.26	++	Additive
---	6	152679729	rs9478326	*SYNE1*	2.25E-06	1.30	0.232	0.189	0.239	0.199	1.9E-05	1.27	0.8464	1.02	0.00013	1.20	++	Additive
YES	6	152681049	rs4286796	*SYNE1*	3.30E-06	1.30	0.221	0.179					*0.9791	*1.00	5.4E-05	1.22	+-	Additive
---	12	9322394	rs75698727	*PZP*	2.92E-08	1.78	0.066	0.038										Additive
---	12	9350854	rs74500107	*PZP*	8.06E-08	1.80	0.060	0.035	0.067	0.039	3.3E-07	1.68	0.3738	0.85	4.7E-05	1.44	+-	Additive
---	12	9358781	rs149632973	*PZP*	4.10E-07	1.75	0.057	0.034	0.055	0.033	2.8E-06	1.70	0.3227	0.82	0.00028	1.43	+-	Additive
---	16	12323566	rs830715	*SNX29*	6.75E-07	1.29	0.300	0.251										Additive
---	10	67559700	rs16921870		7.94E-07	NA	0.094	0.079										Genotypic
**YES**	**10**	**67560490**	**rs10509235**		**8.23E-07**	**NA**	**0.095**	**0.079**					***0.0255**					**Genotypic**
YES	11	5029457	rs11035102	*HBB*	6.93E-08	NA	0.245	0.245					*0.2618					Genotypic
**---**	**11**	**5273865**	**rs113892119**	***HBB***	**5.08E-13**	NA	**0.096**	**0.093**	**0.083**	**0.085**	**3.42E-12**	NA	**0.00178**	**NA**	**NA**	**NA**		**Genotypic**
---	11	5252794	rs12295158	*HBB*	1.32E-10	NA	0.124	0.132	0.123	0.129	2.89E-08	NA	0.2385	NA	NA	NA		Genotypic
**---**	**11**	**5248232**	**rs334**	***HBB***	**1.33E-10**	NA	**0.136**	**0.129**	**0.087**	**0.085**	**4.09E-11**	NA	**0.00077**	**NA**	**NA**	**NA**		**Genotypic**
